# Deciphering the oncogenic network: how C1QTNF1-AS1 modulates osteosarcoma through miR-34a-5p and glycolytic pathways

**DOI:** 10.3389/fonc.2024.1485605

**Published:** 2025-01-09

**Authors:** Yu Zhang, Hailong Lun, Naiqiang Zhu, Ning Yang, Kaikai Ding, Bin Chen, Chengbing Chang, Haipeng Gu, Yanqi Liu

**Affiliations:** ^1^ Graduate School of Chengde Medical University, Chengde, Hebei, China; ^2^ Tangshan Nanhu Hospital, Department of Orthopedic, Tangshan, Hebei, China; ^3^ Hebei Key Laboratory of Panvascular Diseases, Affiliated Hospital of Chengde Medical University, Chengde, Hebei, China; ^4^ Department of Minimally Invasive Spine Surgery, Affiliated Hospital of Chengde Medical University, Chengde, Hebei, China

**Keywords:** OS, C1QTNF1-AS1, miR-34a-5p, LDHA, PDK3, Warburg effect

## Abstract

**Introduction:**

Osteosarcoma (OS), a prevalent metastatic cancer among young individuals, is associated with a grim prognosis. Long non-coding RNAs (lncRNAs), including C1QTNF1-AS1, are pivotal regulators of cancer cell proliferation and motility. As an oncogene, C1QTNF1-AS1 is implicated in various tumor types, such as colorectal, pancreatic, hepatocellular carcinomas, and OS. The aim of this study was to investigate the functions and underlying mechanisms of C1QTNF1-AS1 in the progression of osteosarcoma.

**Methods:**

This investigation focused on elucidating the functional roles and mechanisms of C1QTNF1-AS1 in OS cells. Bioinformatics tools were utilized to identify the interaction between microRNA miR-34a-5p and C1QTNF1-AS1, as well as the targeting of LDHA and PDK3 by miR-34a-5p. Dual-luciferase reporter assays and RNA immunoprecipitation were employed to validate these interactions. Expression profiles of C1QTNF1-AS1, miR-34a-5p, LDHA, and PDK3 in osteosarcoma cells were analyzed using RT-PCR and western blot analyses, revealing their intricate relationships. The impact of these molecules on OS cell proliferation, invasion, and migration was assessed through CCK-8, Transwell, and Cell scratch assay. Moreover, the effects on aerobic glycolysis in OS cells were examined by quantifying ATP levels, lactate production, glucose uptake capacity, and the extracellular acidification rate.

**Results:**

The findings indicated a significant decrease in C1QTNF1-AS1 expression levels in OS cells compared to normal osteoblasts. A parallel downregulation trend of miR-34a-5p was also observed in OS cells. Silencing C1QTNF1-AS1 led to a marked upregulation of LDHA and PDK3 in OS cells, which was partially attenuated by miR-34a-5p mimics. Functional evaluations demonstrated that suppression of C1QTNF1-AS1 accelerated OS cell growth, motility, invasiveness, and the Warburg effect. Conversely, the overexpression of miR-34a-5p mitigated these stimulatory effects, suggesting a regulatory role in modulating OS progression.

**Discussion:**

Our research emphasizes the critical role of C1QTNF1-AS1 in the pathogenesis of osteosarcoma (OS). We discovered that the downregulation of C1QTNF1-AS1 indirectly upregulates the expression of LDHA and PDK3 by suppressing miR-34a-5p, which functions as a regulator of the Warburg effect. This cascade of events promotes OS progression by enhancing glycolytic metabolism and supplying energy for cancer cell growth, migration, and invasion. These findings suggest a potential therapeutic target and highlight the importance of understanding the regulatory network involving lncRNAs in cancer metabolism and progression.

## 1 Introduction

Osteosarcoma, commonly abbreviated as OS, represents a highly aggressive form of connective tissue malignancy characterized by the capability of cancerous cells to produce bone and osteoid tissues (1). It is the leading primary malignancy affecting bone tissues. Posing significant challenges for treatment due to its aggressive nature and propensity for dissemination (2). OS continues to yield poor patient outcomes despite rigorous therapeutic interventions (3). Consequently, there is an urgent need to unravel the intricate molecular mechanisms underpinning OS development, identify novel biomarkers to facilitate early diagnosis, and explore innovative therapeutic strategies to address this formidable disease.

To elucidate the Warburg effect, it implies that cancer cells, under hypoxic conditions, favor lactate fermentation over oxidative phosphorylation for energy generation (4). This metabolic shift is marked by intensified glycolysis, augmented glucose consumption, escalated lactate excretion, and decreased oxygen utilization within tumor cells (5, 6). Extensive research has confirmed the manifestation of the Warburg effect in cancer cells, emphasizing its pivotal contribution to tumor progression and expansion. This phenomenon underscores the metabolic reprogramming of tumor cells, pivotal for their survival and aggressive growth.

PDK3, or pyruvate dehydrogenase kinase isoform 3, serves as a key regulator of intracellular energy metabolism, particularly within tumor cells (7). By inhibiting pyruvate dehydrogenase activity, PDK3 exerts a pronounced influence on the metabolic profile of cancer cells (8). This enzyme plays a central role in perpetuating the Warburg effect, a phenomenon intimately tied to energy metabolism and tumor cell vitality (9). Meanwhile, lactate dehydrogenase A (LDHA), a pivotal player in intracellular lactate dynamics, is intricately linked to both glycolysis and the Warburg effect (10). As a gene and enzyme implicated in glycolysis, LDHA facilitates the generation and accumulation of lactate, thereby bolstering cell survival and proliferation (11). This metabolic pathway is fundamental to cellular energy procurement and, as previous investigations have attested, is a hallmark of OS, underscoring its critical role in the disease.

Long non-coding RNAs (lncRNAs), comprising RNA molecules longer than 200 nucleotides without protein-coding capability, pervade the human genome (12). These transcripts exert substantial influence on the initiation, progression, and development of tumors, either bolstering or retarding these processes (13, 14). For example, FEZF1-AS1, through its interaction with miR-4443, modulates the NUPR1-axis, thereby fostering the development of OS (15). In breast cancer, augmented levels of C1QTNF1-AS1 perturb growth, invasion, and dissemination of cancer cells by regulating various signaling cascades, such as Wnt/β-catenin, PI3K/Akt, and NF-κB (16–19). Likewise, in lung cancer, high expression of C1QTNF1-AS1 orchestrates critical cellular functions, including proliferation, invasion, and angiogenesis, by targeting genes like EGFR, HIF-1α, and VEGF (20–22). However, the precise function of C1QTNF1-AS1 in OS remains an enigma, necessitating further investigation.

MicroRNAs (miRNAs), consisting of approximately 22 nucleotides, are single-stranded, non-coding RNAs that have garnered substantial research attention (23). miR-323a-3p, for instance, amplifies LDHA expression, thereby augmenting lactate generation and fostering metastatic and invasive capabilities in osteosarcoma (24). Conversely, miR-199b-3p retards the expansion of OS by targeting PDK1 (25). Notably, miR-34a-5p exhibits abnormal expression patterns in OS cells, and its levels are intimately tied to cellular responsiveness, tumor stage, lung metastasis potential, and patient prognosis (26, 27).

Competitive endogenous RNA (ceRNA) are RNA molecules that modulate gene expression by interacting with microRNA (miRNA) (28). This class includes various types such as mRNA, lncRNA, pseudogenes, and circRNA (29). The ceRNA mechanism centers on the “sponge effect,” where these molecules bind to miRNA, preventing it from binding to and inhibiting target mRNAs (30, 31). This interaction is based on complementary sequences between ceRNA and miRNA targeting sites (32). The biological significance of ceRNA lies in its role within a gene regulatory network, where ceRNAs compete for miRNAs, creating a complex regulatory system (33). This discovery enhances our comprehension of RNA functions and gene regulation, offering insights into disease mechanisms.

The study unveiled intriguing findings regarding the roles of C1QTNF1-AS1 and miR-34a-5p in OS cells. Specifically, it was observed that both C1QTNF1-AS1 and miR-34a-5p exhibited reduced expression levels in OS cells compared to normal osteoblasts. Furthermore, silencing C1QTNF1-AS1 led to a marked enhancement in OS cell proliferation and the Warburg effect, a phenomenon wherein cancer cells preferentially utilize glycolysis for energy generation even in the presence of oxygen. Bioinformatics analysis illuminated a potential interaction between C1QTNF1-AS1 and miR-34a-5p, suggesting that they partially base pair. Moreover, it was hypothesized that LDHA and PDK3, enzymes implicated in glycolysis and energy metabolism, could be target genes of miR-34a-5p. This interaction was subsequently validated using dual-luciferase reporter assays. The study delved deeper into the underlying mechanisms by exploring whether suppressing C1QTNF1-AS1 indirectly elevated LDHA and PDK3 expression levels through downregulation of miR-34a-5p. This, in turn, was postulated to influence the Warburg effect and accelerate tumor progression.

The research provides valuable insights into the complex interplay between lncRNAs, miRNAs, and their target genes in modulating cancer cell behavior. By shedding light on the regulatory mechanisms governing glycolysis and energy metabolism in OS cells, this study opens new avenues for the development of targeted therapies aimed at disrupting these pathways and ultimately retarding tumor growth and progression.

## 2 Methods

### 2.1 Cell culture and reagents

Human osteosarcoma cell lines (including MG63, Saos-2, U2OS and HOS) and normal osteoblasts (HFOB1.19) were obtained from the ATCC Cell Bank located in Manassas, Virginia, USA. These cells were cultivated in Eagle’s medium supplemented with 10% fetal bovine serum sourced from Thermo Fisher Scientific (Manassas, VA, USA), along with 0.1% penicillin and 0.1% streptomycin procured from Invitrogen (Carlsbad, CA, USA). All osteosarcoma cell lines were cultured at 37°C, whereas the HFOB1.19 cells were maintained at 34°C. All cells were incubated in an environment containing 5% carbon dioxide.

### 2.2 Bioinformatics analysis

The dataset GSE42352 originates from the Gene Expression Omnibus (GEO) database, which is accessible at http://www.ncbi.nlm.nih.gov/geo. The downloaded data comprised 84 disease samples and three healthy controls. The SangerBox platform (http://sangerbox.com) and the limma package (version 3.46.0, Linear Models for Microarray Data, from Bioconductor) were utilized to analyze the two sample groups.

### 2.3 Database analysis

Data pertaining to potential target genes of miR-34a-5p were acquired utilizing the online software tools TarBase (accessible at https://dianalab.e-ce.uth.gr), miRDB (located at https://mirdb.org), and TargetScan (found at https://www.targetscan.org). Analysis conducted with RNAhybrid and miRanda indicated that C1QTNF1-AS1 targets miR-34a-5p. Furthermore, predictions from TargetScan, miRanda, and miRWalk databases suggested that LDHA and PDK3 are potential targets of miR-34a-5p.

### 2.4 Real-time quantitative PCR

In accordance with the manufacturer’s protocol, RNA extraction from osteosarcoma cell lines including MG63, Saos-2, U2OS and HOS was accomplished using the TRIzol reagent sourced from Thermo Fisher Scientific. Subsequently, the RNA samples were reverse transcribed into cDNA using the PrimeScript RT Kit provided by Takara. Gene expression levels were then quantified via qRT-PCR on an ABI7500 Quantitative PCR instrument from ABI Corporation, employing the SYBR Prex Ex Taq II Kit also from Takara. GAPDH served as the stable reference gene for normalization during the analysis.

### 2.5 Western blotting

Total protein extraction was carried out using RIPA buffer (Sigma) supplemented with a protease inhibitor from Roche. The extracted proteins were then resolved on a 12% SDS-PAGE gel. After electrophoresis, the proteins were transferred to membranes which were subsequently blocked with 5% skim milk for an hour. Overnight incubation with the primary antibody at 4°C was followed by rinsing the membranes with TBST. Subsequently, the membranes were incubated with secondary antibodies and washed again with TBST. Luminescence was detected using an ECL detection kit from Share-bio. For quantification, densitometric analysis of the immunoblotted proteins was conducted utilizing ImageJ software.

### 2.6 Cell count kit-8 test

Following transfection, U2OS and MG63 cells were plated in 96-well plates at a concentration of 3,000 cells per well. Each well received 10μL of CCK-8 solution (Dojindo Molecular Technologies) and was incubated at 37°C with 5% CO2 for 0, 24, and 48 hours. The optical density at 450 nm was then determined using a BioTek microplate reader. The findings reflect three separate trials.

### 2.7 Cell transfection

Cells with C1QTNF1-AS1 knockdown (si-lnc) and those with a negative control (si-NC) were produced. GenePharma (Shanghai, China) supplied the miR-34a-5p mimic (miR-mim) along with its control (miR-NC). Lipofectamine 3000 reagent (Invitrogen) was utilized for transfection, following the guidelines provided by the manufacturer.

### 2.8 Measurement of glucose metabolism, lactate production, and intracellular ATP levels

The rate of glucose metabolism was assessed using the colorimetric glucose uptake test kit provided by Sigma-Aldrich. The determination of intracellular ATP concentration was carried out according to the protocol provided by Promega Corporation (Madison, Wisconsin, USA), using their ATP Assay Kit. Fluorescence measurements of bioluminescence were taken using a fluorometer produced by Perkin Elmer located in Waltham, Massachusetts, USA. ATP levels were calculated based on the standard curve method. Furthermore, the extracellular lactate levels were measured utilizing a lactate assay kit sourced from BioVision (Zurich, Switzerland), in accordance with the manufacturer’s protocol. All measurements were normalized to the cellular protein levels. The experiment was independently replicated three times.

### 2.9 Transwell assay

A matrix-coated 24-well span chamber (8μm aperture) was prepared for cell invasion assays. U2OS and MG63 cells were incubated in a serum-free medium in the upper chamber. The lower chamber received medium with 10% FBS. Following a 48-hour incubation period, the cells that had migrated to the lower chamber were treated with methanol and stained using a 0.1% crystal violet solution. The invading cells were examined using an Olympus inverted microscope.

### 2.10 Cell scratch assay

Cell scratch tests were utilized to evaluate the movement of OS cell lines. Once the U2OS and MG63 cells achieved 90% confluence in 24-well plates, a sterile plastic tip was used to scrape the monolayer, followed by two washes with phosphate-buffered saline (PBS) to eliminate cell debris. The cells were then incubated in complete growth medium. Ultimately, the cells that moved to the damaged region were gathered at 0 and 24 hours following the initial scratch and examined using an inverted microscope (Olympus) for each injury. The relative distances of the cell scratches were analyzed using ImageJ software.

### 2.11 Dual-luciferase activity measurement

A dual-luciferase reporter assay was conducted by inserting either wild-type or mutant lncRNA-C1QTNF1-AS1/LDHA/PDK3 into the pmirGLO vector (Universal Biotech, China). MG63 cells were planted in 48-well plates at a concentration of 5×104 cells per well. For 48 hours, the luciferase reporter plasmid along with miR-34a-5p mimic or mimic-NC were introduced using Liposome 3000 (Invitrogen). The activities of firefly and Renilla luciferase were evaluated using a dual-luciferase reporter assay kit from Promega, USA. The activity of Firefly luciferase was adjusted relative to that of Renilla luciferase. Every test was conducted three times.

### 2.12 Measurement of glycolysis

The extracellular acidification rate (ECAR) of cultured cells was measured using an XF96 metabolic flux analyzer from Seahorse Biosciences, located in Billerica, MA, USA, in accordance with the manufacturer’s instructions. Briefly, 80 µL of a suspension containing 3 × 10^4 target cells were dispensed into each well of an XF96 96-well plate provided by Seahorse Biosciences and incubated overnight at 37°C. The XF sensor box, manufactured by Seahorse Bioscience, was prepared with XF calibration solution and left to incubate overnight at 37°C, without the addition of CO2. On the subsequent day, the standard medium was replaced with XF assay-adapted DMEM (containing 1 g/mL glucose, pH 7.4; sourced from Seahorse Biosciences), and the cells were further incubated for an hour at 37°C in a CO2-free environment. Sequential additions of 10 mM glucose, 1 mM oligomycin (provided by Sigma-Aldrich), and 80 mM 2-deoxyglucose (D8375; Sigma-Aldrich) were made to assess ECAR. The obtained data were analyzed using XFe Wave software, also provided by Seahorse Biosciences.

### 2.13 RNA immunoprecipitation assay

This study employed the RIP Kit manufactured by Bersinbio Company (Guangzhou, China), adhering strictly to the manufacturer’s operating instructions, to assess the interactions among LDHA, PDK3, miR-34a-5p, and the AGO2 protein. During the experimental procedure, cells subjected to oxidative stress were initially washed with phosphate-buffered saline (PBS). Following this, cell lysis was conducted in RIP buffer supplemented with proteasome inhibitors. The resultant lysate was then co-incubated overnight with antibodies specifically targeting the AGO2 protein or with control IgG antibodies, to facilitate the formation of RNA-protein complexes. Subsequently, these complexes were bound to protein A/G magnetic beads and subjected to proteinase K treatment to eliminate the protein components. Ultimately, the purified RNA was analyzed using qRT-PCR.

### 2.14 Statistical analysis

Data were analyzed and graphed using GraphPad Prism 9 (version 9.4.0). Data are presented as mean ± standard deviation, and group differences were analyzed using the t-test Or the One-way test. A p-value of less than 0.05 was considered statistically significant.

## 3 Results

### 3.1 C1QTNF1-AS1 expression was significantly downregulated in OS cells

**Figure 1:**
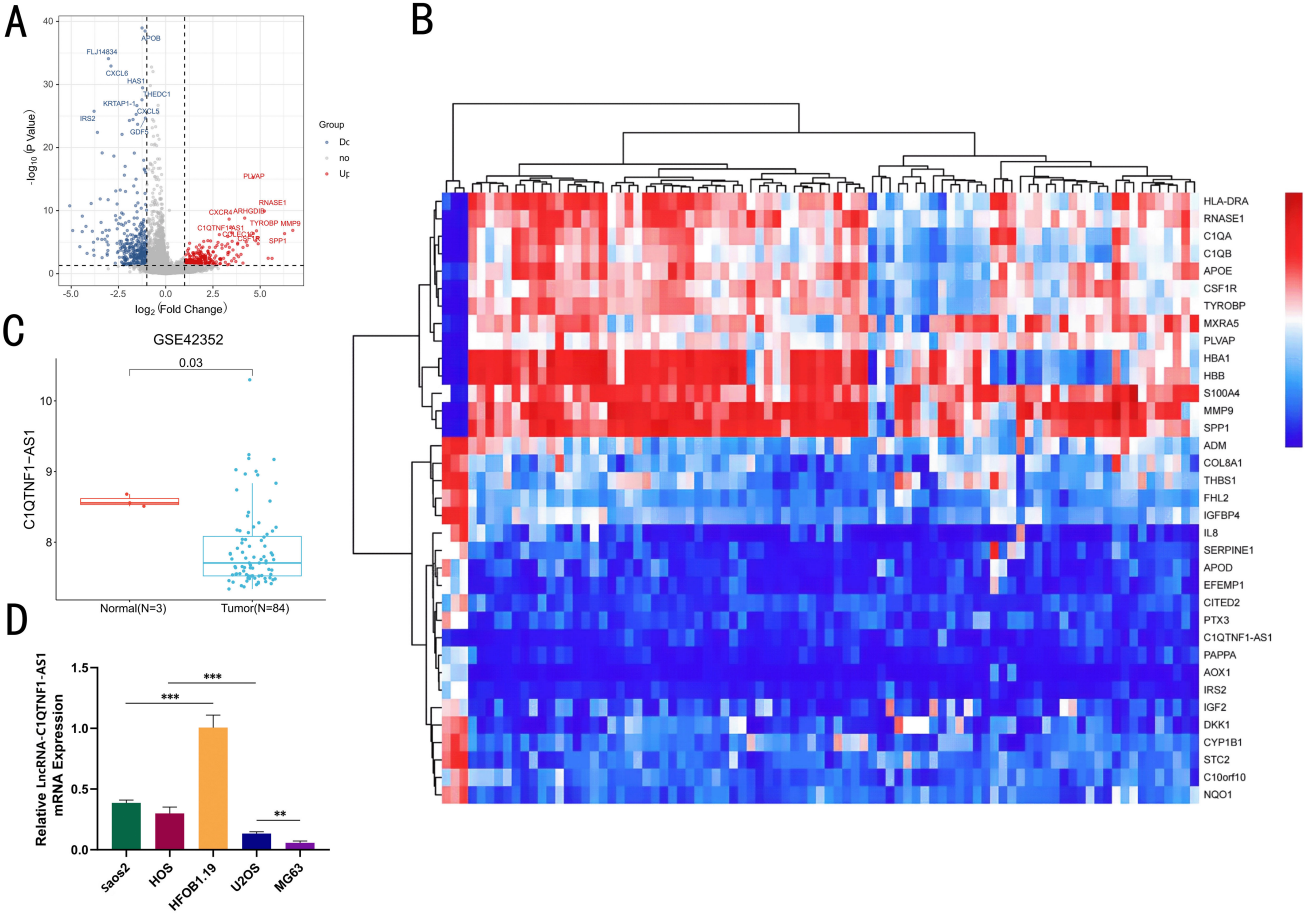
The levels of LncRNA‐C1QTNF1-AS1 are reduced in OS. A volcano plot showcasing the LncRNA with differential expression in GEO datasets **(A)**. A heatmap illustrating the variably expressed LncRNA in GSE42352, sourced from the GEO database **(B)**. Expression of LncRNA C1QTNF1-AS1 in normal cells and OS cells of dataset **(C)**. Quantitative real-time PCR **(D)** was employed to measure the levels of LncRNA-C1QTNF1-AS1 in U2OS, Saos2, MG63, and HOS cells compared to hFOB1.19 cells. Results are shown as average ± standard deviation. **P<0.01, ***P<0.001.

The SangerBox platform was utilized to analyze gene expression profiles from the GSE42352 dataset on GEO, aiming to pinpoint genes with differential expression in OS cells. Differentially expressed genes between OS cells and normal osteoblasts were identified using the limma package (log FC > 1 and p < 0.05) and volcano and heatmap plots were prepared in The R Project for Statistical Computing (R version 4.4.0) (**Figures 1A, B**). Among these differentially expressed genes, C1QTNF1-AS1 was significantly downregulated in OS cells compared to that in normal osteoblasts (**Figure 1C**). Initially, we assessed the expression levels of C1QTNF1-AS1 in both osteoblasts (HFOB 1.19) and various osteosarcoma cell lines (Saos2, MG63, HOS, and U2OS) through qRT-PCR analysis. The findings indicated that C1QTNF1-AS1 expression was notably reduced in osteosarcoma cell lines relative to normal human osteogenic cell lines, with a marked decrease observed in MG63 and U2OS cell lines (**Figure 1D**).

### 3.2 Silencing of C1QTNF1-AS1 significantly promoted OS cell development and the Warburg effect *in vitro*

**Figure 2:**
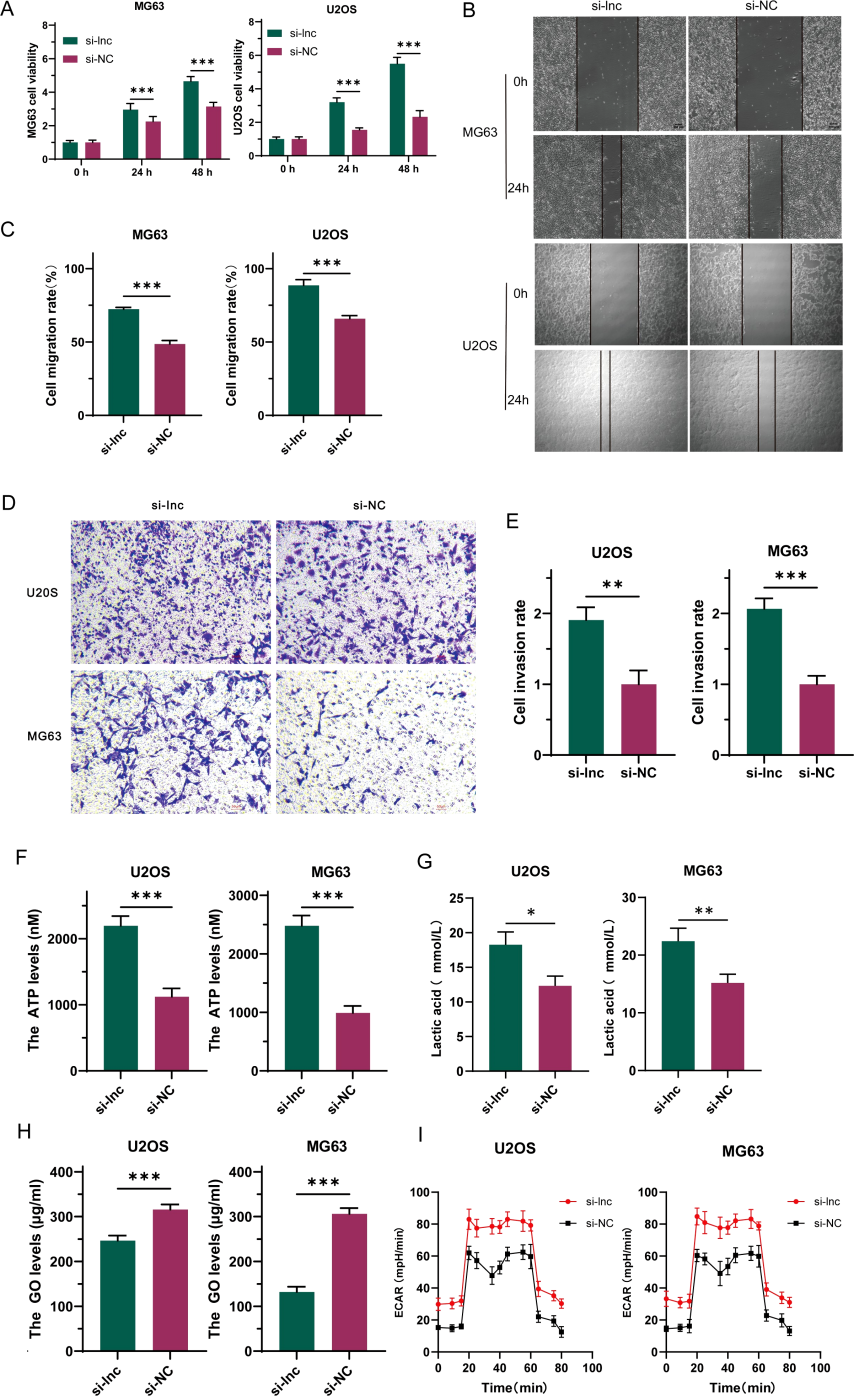
LncRNA-C1QTNF1-AS1 exhibits inhibitory effects on the progression of osteosarcoma (OS) in laboratory conditions. The CCK-8 assay was utilized to evaluate the growth rate of OS cells following the inhibition of C1QTNF1-AS1 **(A)**. The migration capability of cells after C1QTNF1-AS1 knockdown was assessed through a cell scratch assay **(B, C)**. The invasive potential post-inhibition of C1QTNF1-AS1 was further examined using a Transwell assay **(D, E)**. To gain insights into the metabolic alterations, ATP **(F)** and lactate production levels **(G)** in C1QTNF1-AS1 knockdown cells were measured using respective assays, while glucose concentrations in cell supernatants were determined via a glucose assay **(H)**. To definitively determine the impact of C1QTNF1-AS1 silencing on glycolysis rates, the extracellular acidification rate (ECAR) was assessed using a metabolic flux analyzer **(I)**. Results are shown as average ± standard deviation. *P<0.05, **P<0.01, ***P<0.001.

To investigate the precise role of C1QTNF1-AS1 in osteosarcoma cells, we created a cell line with C1QTNF1-AS1 knocked down (si-lnc) and a corresponding negative control (si-NC). The results of the CCK-8 proliferation assay (**Figure 2A**), cell scratch tests (**Figures 2B, C**), along with the Transwell experiment (**Figures 2D, E**) demonstrated that reducing C1QTNF1-AS1 levels enhanced the growth, movement, and invasive capabilities of MG63 and U2OS cells. The Warburg effect is essential for tumor progression and aids in the proliferation of cancer cells. We investigated the potential correlation between C1QTNF1-AS1 and the Warburg effect in the development of osteosarcoma (OS) by examining glucose levels, ATP production, and lactate production in the supernatants of OS cell cultures. To ascertain whether the silencing of C1QTNF1-AS1 contributes to the regulation of aerobic glycolysis, we utilized a metabolic flux analyzer to measure extracellular acidification rates (ECAR). Our research revealed that the silencing of C1QTNF1-AS1 led to a substantial increase in ATP production (**Figure 2F**) and lactate production (**Figure 2G**) in MG63 and U2OS cells. Simultaneously, it notably decreased the glucose levels in the supernatants of these cells (**Figure 2H**), indicating that the cells consumed more glucose. Furthermore, we found that the knockdown of C1QTNF1-AS1 resulted in enhanced glycolysis levels in U2OS and MG63 cells (**Figure 2I**). Taken together, these data suggest that the silencing of C1QTNF1-AS1 promotes aerobic glycolysis in OS cells.

### 3.3 miR-34a-5p was identified as a direct target of C1QTNF1-AS1 and showed consistent expression trends in OS cells

**Figure 3:**
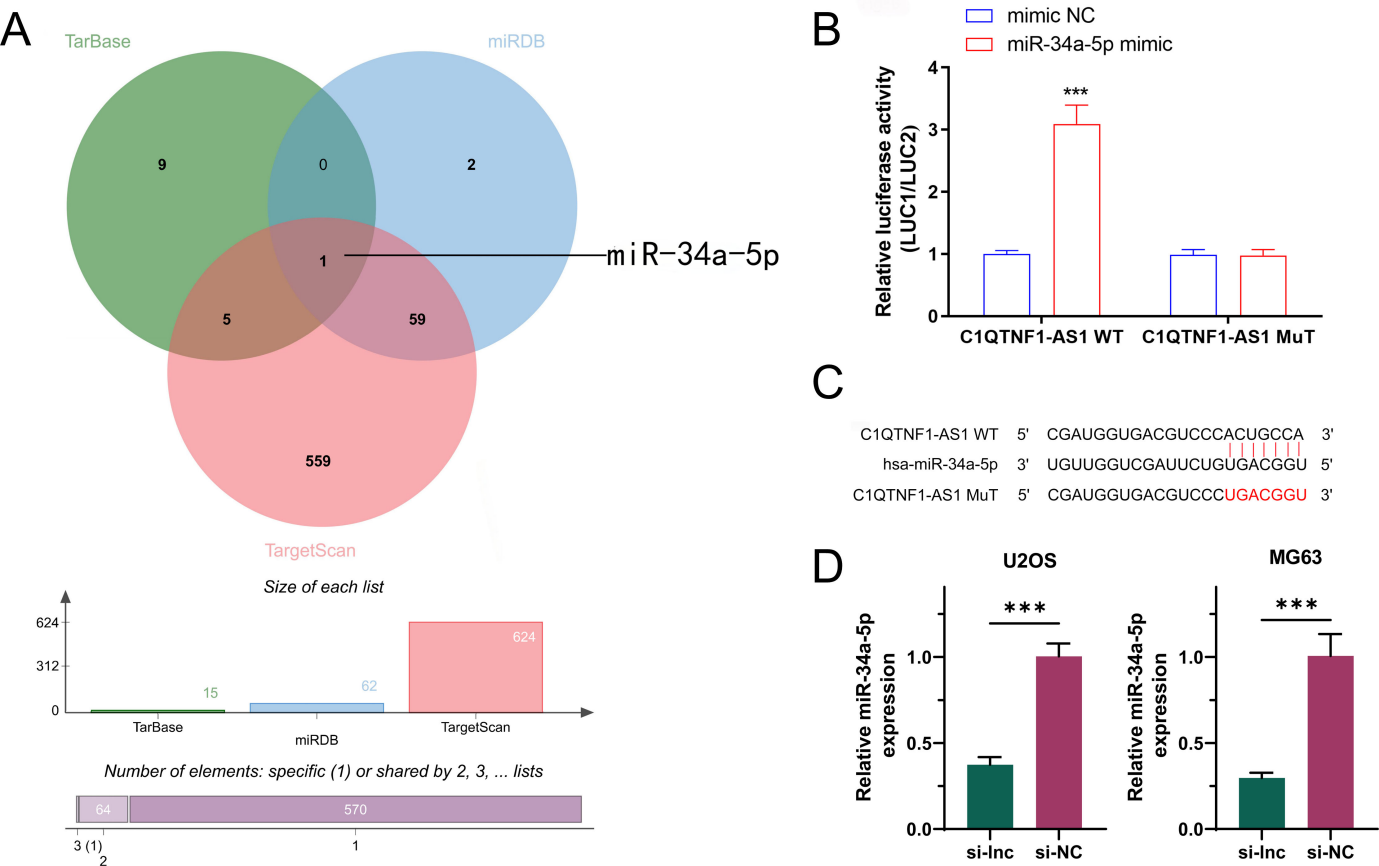
miR-34a-5p is an authentic target of LncRNA‐C1QTNF1-AS1.A Venn diagram illustrating the anticipated glycolysis-associated target genes of lncRNA C1QTNF1-AS1, derived from the TarBase miRDB, and TargetScan databases **(A)**. Dual-luciferase reporter experiments were conducted on OS cells transfected with either miR-34a-5p UTR wild-type (WT) or mutant (MUT) forms, alongside lncRNA C1QTNF1-AS1 plasmid or a control plasmid **(B)**. miR-34a-5p 3′‐UTR contains one predicted LncRNA C1QTNF1-AS1 binding site **(C)**. Expression levels of miR-34a-5p in OS cells after transfection with either a negative control or a C1QTNF1-AS1 knockdown plasmid **(D)**. Results are shown as the average ± standard deviation. Results are shown as average ± standard deviation. ***P<0.001.

In order to investigate how C1QTNF1-AS1 influences aerobic glycolysis in OS, we employed three gene prediction tools—TarBase, miRDB, and TargetScan—to identify mRNA candidates targeted by C1QTNF1-AS1.Intersection of the three databases revealed that only miR-34a-5p was associated with aerobic glycolysis (**Figure 3A**). The binding site of C1QTNF1-AS1 to miR-34a-5p was predicted using the RNAhybrid and miRanda algorithms, and a mutated sequence, mut, was designed. The dual-luciferase assay results showed that the miR-34a-5p mimic could bind to C1QTNF1-AS1 wild-type to increase luciferase activity compared to the expression of the NC mimic. Nonetheless, altering the binding site resulted in the miR-34a-5p mimic showing no notable impact on luciferase activity (**Figure 3B**). This suggested that miR-34a-5p attaches to C1QTNF1-AS1 at this location (**Figure 3C**). In order to investigate the connection between C1QTNF1-AS1 and miR-34a-5p in osteosarcoma cells, we created a stable cell line (si-lnc) and a negative control (si-NC). The qRT-PCR analysis indicated that miR-34a-5p levels in osteosarcoma cells decreased following the silencing of C1QTNF1-AS1 (**Figure 3D**).

### 3.4 miR-34a-5p suppressed the growth and progression of OS cells by modulating the Warburg effect

**Figure 4:**
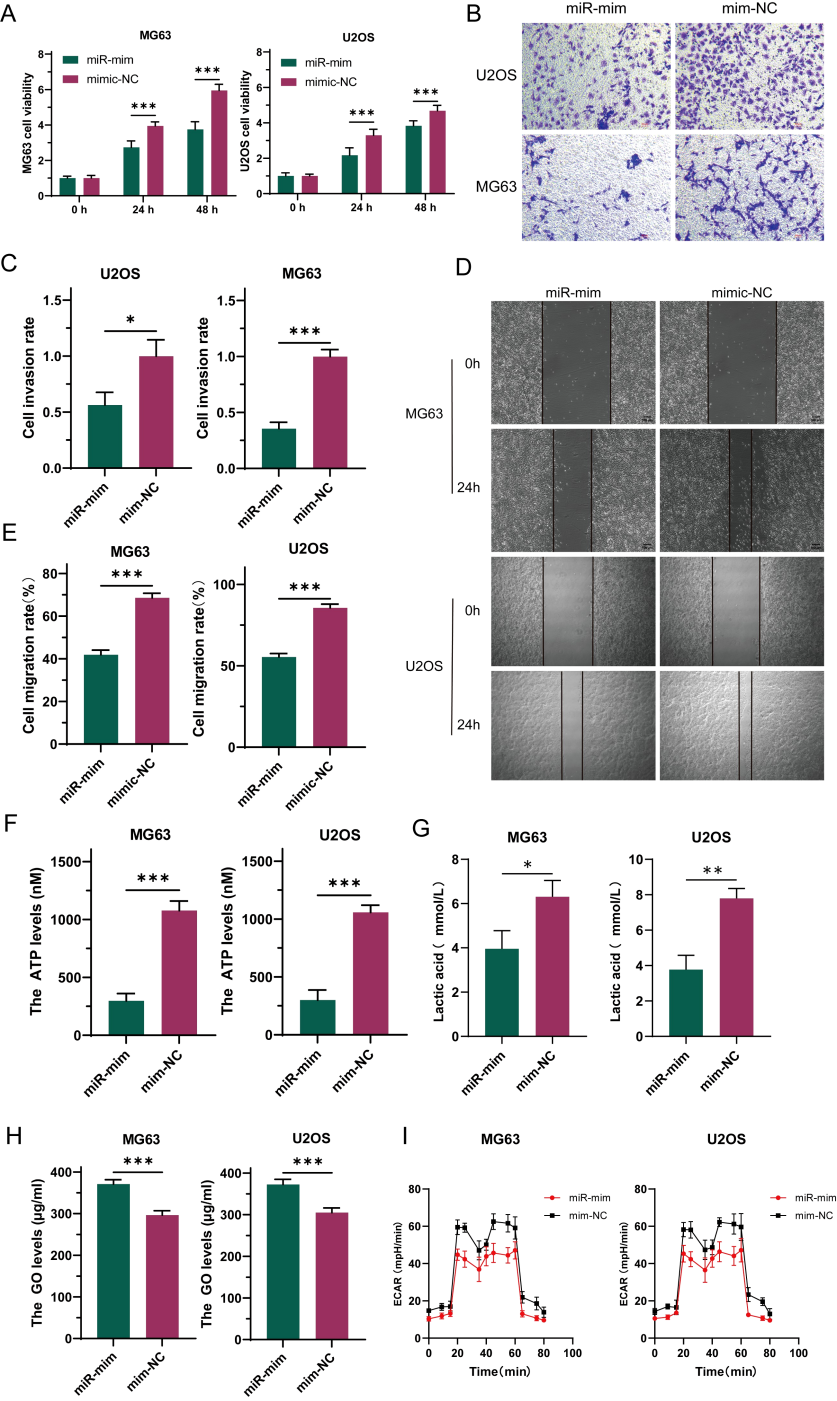
Under laboratory conditions, miR-34a-5p has been demonstrated to inhibit the progression of osteosarcoma (OS). To investigate this, the proliferation rate of OS cells was assessed following miR-34a-5p overexpression using CCK-8 assays **(A)**. Furthermore, the invasive potential of these cells with miR-34a-5p overexpression was evaluated through Transwell experiments **(B, C)**. To determine the effect of miR-34a-5p overexpression on cell migration, scratch assays were conducted on the cells **(D, E)**. Additionally, to assess the production of ATP and lactate in cells overexpressing miR-34a-5p, ATP levels **(F)**, lactate levels **(G)**, and glucose content in the cell supernatant **(H)** were measured using appropriate assays. Moreover, ECAR detection was performed to evaluate the glycolytic rate of the cells after miR-34a-5p overexpression **(I)**. Results are shown as average ± standard deviation. *P<0.05, **P<0.01, ***P<0.001.

To explore the function of miR-34a-5p in osteosarcoma, this study constructed stable cell lines overexpressing miR (miR-mim) and negative controls (mim-NC) and performed CCK-8 proliferation assays. The results showed that increased expression levels of miR-34a-5p inhibited the growth of OS cells (**Figure 4A**). Transwell assay results indicated that elevated miR-34a-5p expression levels suppressed the invasion of OS cells (**Figures 4B, C**). Scratch assays demonstrated that overexpression of miR-34a-5p inhibited the migration of OS cells (**Figures 4D, E**). This study found that the overexpression of miR-34a-5p significantly reduced ATP production (**Figure 4F**) and lactate production (**Figure 4G**) in MG63 and U2OS cell lines, while significantly increasing the glucose level in the cell supernatant (**Figure 4H**). This indicated a reduction in glucose consumption by osteosarcoma cells. Metabolic flux analysis revealed that the overexpression of miR-34a-5p significantly decreased the extracellular acidification rate (ECAR) (**Figure 4I**) in both MG63 and U2OS cell lines, thereby inhibiting the Warburg effect. In summary, miR-34a-5p inhibited the proliferation, migration, and invasion of osteosarcoma cells, as well as aerobic glycolysis.

### 3.5 Silencing of C1QTNF1-AS1 promoted OS progression through miR-34-a-5p–mediated glycolysis

**Figure 5:**
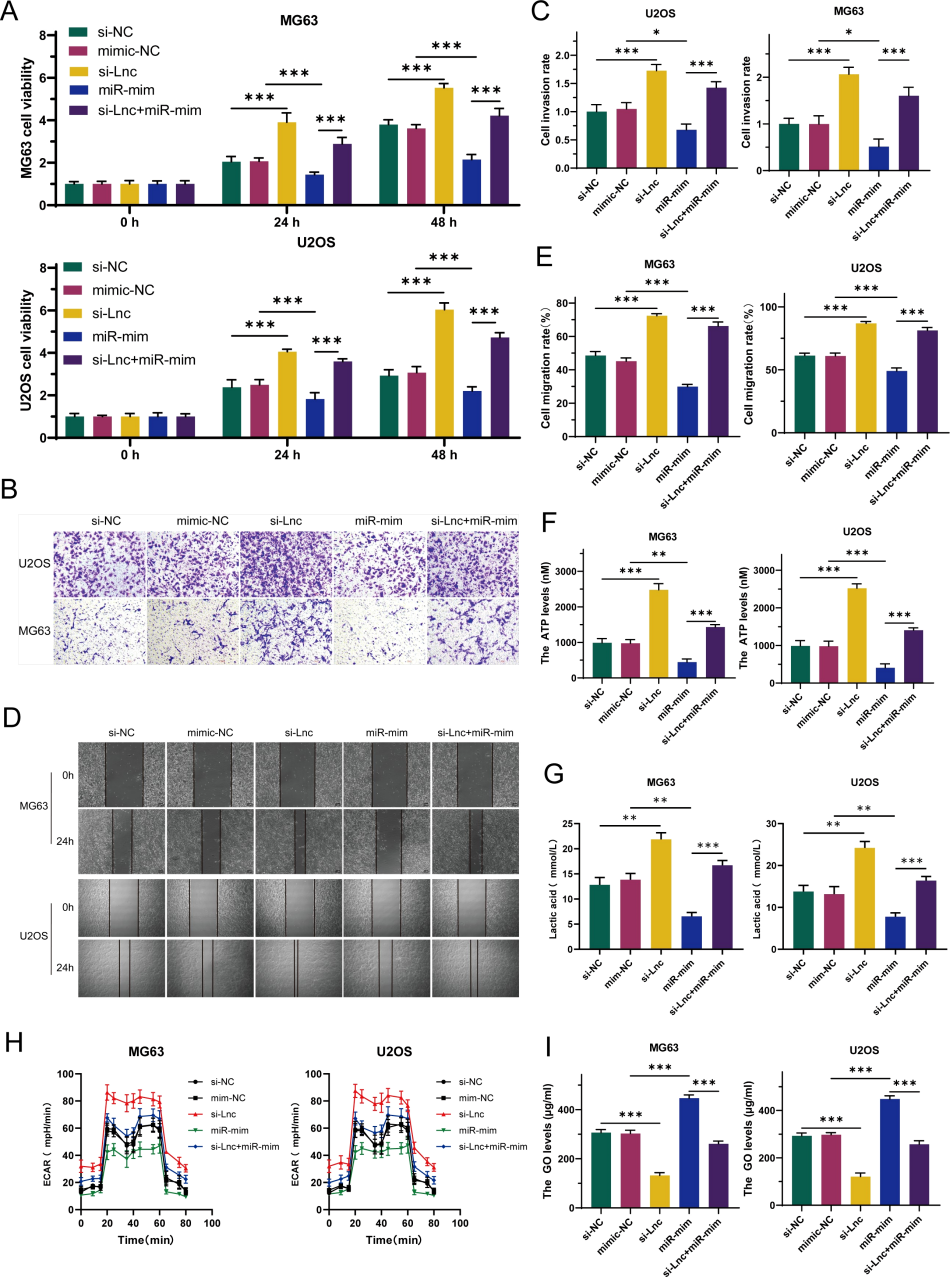
Elevated levels of miR-34a-5p partially restore the oncogenic role suppressed by the downregulation of C1QTNF1-AS1 in osteosarcoma. CCK-8 tests **(A)**, Transwell assays **(B, C)**, and cell scratch tests **(D, E)** demonstrate that the overexpression of miR-34a-5p somewhat counteracts the enhancement of OS cell proliferation, invasion, and migration induced by the knockdown of C1QTNF1-AS1. Furthermore, assessments of ATP levels **(F)**, lactate production **(G)**, glucose consumption **(H)**, and ECAR **(I)** indicate that the overexpression of miR-34a-5p also somewhat mitigates the impact of C1QTNF1-AS1 silencing on the Warburg effect in osteosarcoma cells. Results are shown as average ± standard deviation. *P<0.05, **P<0.01, ***P<0.001.

To deeply explore whether C1QTNF1-AS1 inhibits the development and progression of osteosarcoma cells by targeting miR-34a-5p, this study selected U2OS and MG63 cell lines for rescue experiments. By comparing the data from CCK-8 assays (**Figure 5A**), Transwell assays (**Figures 5B, C**), and cell scratch assays (**Figures 5D, E**), it was found that knockdown of C1QTNF1-AS1 significantly promoted the proliferation, migration, and invasion abilities of osteosarcoma cells, while the overexpression of miR-34a-5p partially reversed this effect. Similarly, the elevation of miR-34a-5p levels partially alleviated the impact of C1QTNF1-AS1 inhibition on the Warburg effect of osteosarcoma cells. Further confirmation of this effect was provided by measuring ATP production (**Figure 5F**), lactate production (**Figure 5G**), glucose consumption (**Figure 5H**), and ECAR (**Figure 5I**). Based on these results, this study revealed that inhibiting C1QTNF1-AS1 enhances the proliferation, migration, and invasion abilities of osteosarcoma cells by blocking the glycolysis driven by miR-34a-5p.

### 3.6 LDHA and PDK3 were identified as direct targets of miR-34a-5p and showed opposite expression trends in OS cells

**Figure 6:**
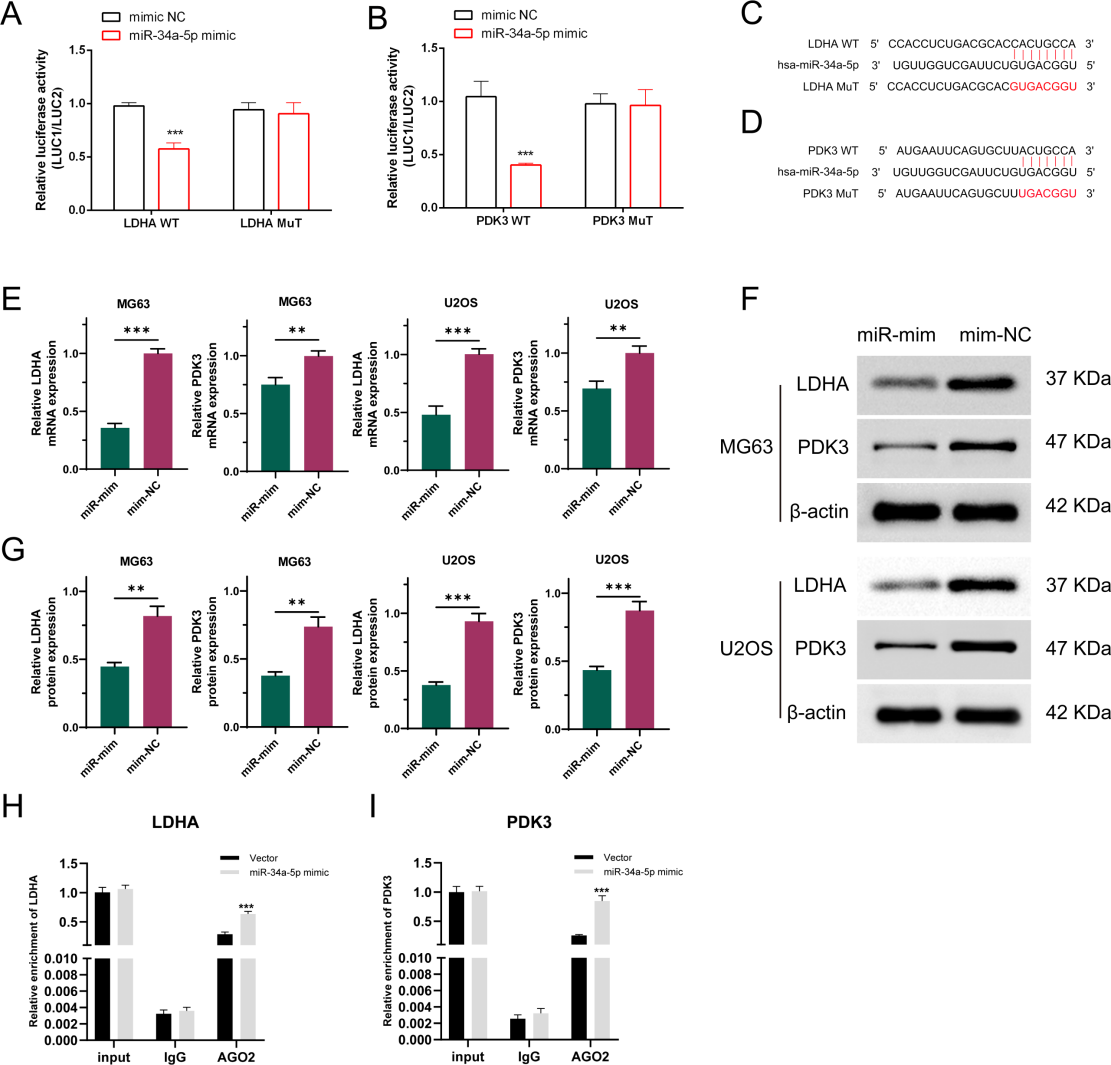
LDHA and PDK3 are authentic targets of miR-34a-5p. Dual-luciferase reporter experiments were conducted on OS cells transfected with either wild-type or mutant LDHA and PDK3 UTR, alongside miR-34a-5p plasmid or a control plasmid **(A, B)**. The 3′-UTRs of LDHA and PDK3 both have a predicted binding site for miR-34a-5p **(C, D)**. As demonstrated by qRT-PCR **(E)** and Western blot analyses **(F, G)**, the expression levels of LDHA and PDK3 were assessed in osteosarcoma cells transfected with either a negative control plasmid or a plasmid overexpressing miR-34a-5p. RIP experiments were conducted using MG63 cell lysates with AGO2 or IgG antibodies. qRT-PCR results showed the relative enrichment of LDHA and PDK3 mRNA in cells transfected with miR-34a-5p mimics compared to those transfected with NC mimics **(H, I)**. Results are shown as average ± standard deviation. **P<0.01, ***P<0.001.

The binding sites for LDHA, PDK3, and miR-34a-5p were predicted utilizing the TargetScan, miRanda, and miRWalk algorithms, and a mutated sequence (mut) was subsequently designed. The dual-luciferase assay revealed that the miR-34a-5p mimic attached to both wild-type LDHA and PDK3, resulting in a reduction in luciferase activity compared to the NC mimic expression. However, mutating the binding site rendered the miR-34a-5p mimic ineffective in altering luciferase activity (**Figures 6A, B**). These findings demonstrated that miR-34a-5p binds to LDHA and PDK3 at the predicted sites (**Figures 6C, D**). Furthermore, stable cell lines overexpressing miR-34a-5p (miR-mim) and a negative control (mim-NC) were established. The qRT-PCR analysis results indicated that the expression levels of both PDK3 and LDHA were significantly downregulated in osteosarcoma cells following the overexpression of miR-34a-5p (**Figure 6E**). Western blot analysis further confirmed this observation, demonstrating a significant decrease in the protein expression of PDK3 and LDHA in osteosarcoma cells due to the overexpression of miR-34a-5p (**Figures 6F, G**). RNA immunoprecipitation (RIP) experiments revealed that the enrichment levels of LDHA and PDK3 were significantly increased in the group overexpressing miR-34a-5p and targeting Argonaute2 (Ago2) (**Figures 6H, I**). These experimental data collectively demonstrated that miR-34a-5p inhibited the expression of LDHA and PDK3 by directly interacting with their 3′ untranslated regions (3′UTR).

### 3.7 Silencing of C1QTNF1-AS1 upregulates LDHA and PDK3 expression in OS cells through inhibition of miR-34a-5p

**Figure 7:**
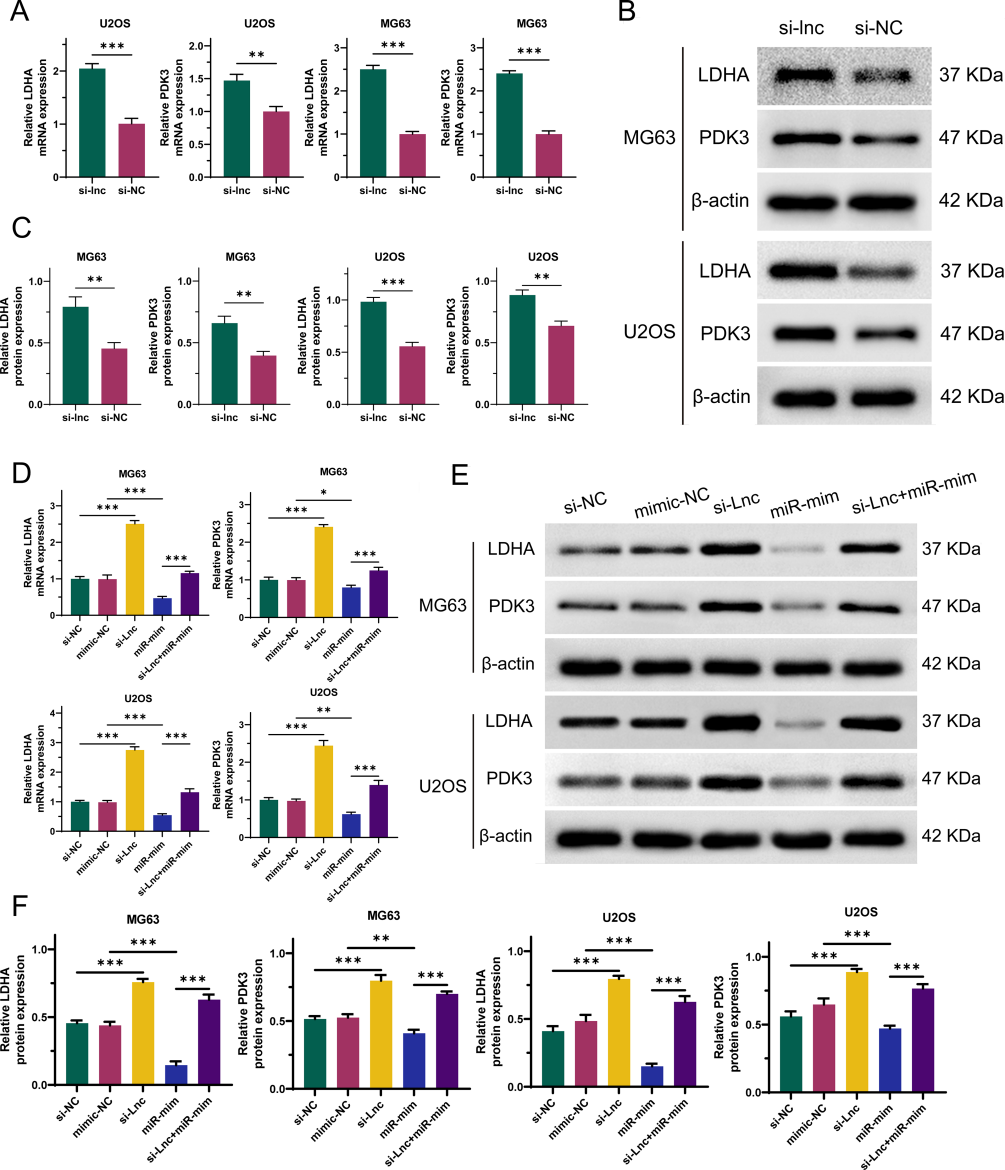
Silencing of C1QTNF1-AS1 upregulates the expression of LDHA and PDK3 in osteosarcoma (OS) cells by inhibiting miR-34a-5p. The altered expression levels of LDHA and PDK3 in OS cells subsequent to C1QTNF1-AS1 silencing are demonstrated through qRT-PCR **(A)** and Western blot analysis **(B, C)**. As illustrated by qRT-PCR **(D)** and Western blot analysis **(E, F)**, overexpression of miR-34a-5p partially reverses the enhanced expression of LDHA and PDK3 that is observed following the knockdown of C1QTNF1-AS1. Results are shown as average ± standard deviation. *P<0.05, **P<0.01, ***P<0.001.

To explore the interactions between C1QTNF1-AS1, miR-34a-5p, LDHA, and PDK3 in OS cells, we constructed C1QTNF1-AS1 knockdown stable cell lines (si-lnc) and miR-34a-5p overexpression stable cell lines (miR-mim). As demonstrated by qRT-PCR (**Figure 7A**) and Western blot analyses (**Figures 7B, C**), the suppression of C1QTNF1-AS1 in OS cells led to a notable rise in LDHA and PDK3 levels. As demonstrated by qRT-PCR (**Figure 7D**) and Western blot analyses (**Figures 7E, F**), our rescue experiments revealed that elevating miR-34a-5p levels partially mitigated the enhancement induced by LDHA and PDK3 following the knockdown of C1QTNF1-AS1.

## 4 Discussion

OS, a common form of primary bone malignancy encountered in clinical practice, predominantly strikes adolescents and children and boasts the highest incidence rate among its kind. Characterized by rapid progression and a high tendency for metastasis (34). OS is considered a highly aggressive cancer. In the realm of cancer development, long non-coding RNAs (lncRNAs) occupy a pivotal position (35). This particular study delves into the functional roles and underlying mechanisms of the lncRNA, C1QTNF1-AS1, in the context of osteosarcoma. The findings of this investigation reveal that the inhibition or silencing of C1QTNF1-AS1 leads to an upregulation of LDHA and PDK3 expressions, a phenomenon mediated through the sequestration of miR-34a-5p. This observation underscores the intricate interplay between various regulatory elements within the cancer cell landscape.

The provided text discusses the role of C1QTNF1-AS1 in various types of cancer, including OS. It highlights that C1QTNF1-AS1 expression is reduced in certain cancer cells and that it inhibits multiple types of cancer by affecting proliferation, invasion, epithelial-mesenchymal transition, and apoptosis induction (36, 37). Additionally, C1QTNF1-AS1 counteracts the Warburg effect in hepatocellular carcinoma (38). The study confirms the function of C1QTNF1-AS1 in OS cells, showing that silencing C1QTNF1-AS1 promotes proliferation, migration, invasion, and the Warburg effect in OS cells. Overall, the findings indicate that C1QTNF1-AS1 is essential in the advancement of OS.

miR-34a-5p inhibits Thyroid cancer progression by restricting cell growth and spread and also hampers Head and neck squamous cell carcinoma development by targeting Flotillin-2 (39). In this research, miR-34a-5p was identified as a target of C1QTNF1-AS1, confirmed through dual-luciferase reporter tests. Our analysis revealed a significant association between the levels of miR-34a-5p and C1QTNF1-AS1 in OS cells. Functional assays confirmed that miR-34a-5p overexpression suppresses cell growth, movement, invasion, and the Warburg effect in OS cells. Rescue experiments showed that silencing C1QTNF1-AS1 led to increased OS cell growth, invasion, migration, and the Warburg effect by sequestering miR-34a-5p. These data indicate that C1QTNF1-AS1 regulates OS progression by adsorbing miR-34a-5p.

The Warburg effect plays a pivotal role in fostering the growth and progression of malignant tumors (40). This effect reveals a biological phenomenon wherein, despite ample oxygen availability, osteosarcoma cells preferentially obtain energy through glycolysis rather than through the normal oxidative phosphorylation pathway (41). This metabolic pattern not only provides the necessary energy and biosynthetic precursors for rapid tumor cell proliferation but may also promote tumor invasion and metastasis by altering the tumor microenvironment (42). The presence of the Warburg effect offers new avenues for osteosarcoma treatment. Firstly, researchers can target key enzymes in the glycolytic pathway, such as hexokinase and lactate dehydrogenase, to develop specific inhibitors. These inhibitors can effectively suppress the energy metabolism of tumor cells, thereby inhibiting their growth (43). Secondly, by exacerbating mitochondrial dysfunction and promoting mitochondria-mediated apoptosis, the survival capacity of tumor cells can be further compromised (44). Additionally, intervention in the Warburg effect may synergize with existing chemotherapeutic agents, enhancing the sensitivity of tumor cells to drugs and thus intensifying the efficacy of chemotherapy (45). The development of these therapeutic strategies not only promises to improve the survival rates of osteosarcoma patients but may also enhance their quality of life. Through in-depth investigation of the relationship between the Warburg effect and osteosarcoma, scientists are progressively unveiling the mechanisms of aberrant tumor metabolism, laying a solid foundation for future personalized and precision medicine approaches. With ongoing research, there is good reason to believe that further breakthroughs in the field of osteosarcoma treatment will be achieved, offering more hope and possibilities for patients.

Notably, lactate dehydrogenase A (LDHA), a pivotal enzyme in the final phase of this metabolic shift, is prevalent in multiple cancer cells and is intimately linked to tumor dimensions and clinical outcomes (46, 47). Pyruvate dehydrogenase kinase (PDK), with its four isoforms, contributes significantly to the emergence of the Warburg effect and balances glycolysis with oxidative phosphorylation (48, 49). Our study pinpointed LDHA and PDK3 as direct targets of microRNA-34a-5p (miR-34a-5p), validated through dual-luciferase reporter assays and RNA immunoprecipitation. Additionally, analyzing the expression patterns of C1QTNF1-AS1, miR-34a-5p, LDHA, and PDK3 in OS cells, along with rescue experiments, revealed that the silencing of C1QTNF1-AS1 upregulates LDHA and PDK3 levels in OS cells by sequestering miR-34a-5p.

In summary, this study elucidates the potential biomarker value of C1QTNF1-AS1 and miR-34a-5p in the early diagnosis of osteosarcoma. By quantitatively analyzing the expression levels of these two molecules in patients, early identification and diagnosis of osteosarcoma can be achieved, thereby facilitating timely and effective therapeutic interventions aimed at improving patient survival rates. Furthermore, the study reveals the unique metabolic characteristics of osteosarcoma cells, namely the Warburg effect. This research provides a novel perspective for the formulation of treatment strategies: 1. The restoration or enhancement of miR-34a-5p function can effectively inhibit the proliferation and invasiveness of osteosarcoma cells, and by modulating the expression of enzymes such as LDHA and PDK3, the metabolic profile of osteosarcoma cells can be altered. This lays the theoretical groundwork for the development of miR-34a-5p-targeted therapeutic approaches, potentially representing a novel method for treating osteosarcoma. 2. Suppressing the expression of LDHA and PDK3 *in vivo* can disrupt the metabolic pathways of osteosarcoma cells, thereby inhibiting their proliferation and invasiveness. This provides a basis for the development of targeted drugs against LDHA and PDK3. 3. Combining miR-34a-5p-targeted therapies with current conventional treatments for osteosarcoma may yield superior therapeutic outcomes. By restoring miR-34a-5p function or inhibiting LDHA and PDK3 activity, patient sensitivity to chemotherapy or radiotherapy can be enhanced, thus improving treatment efficacy and reducing side effects. In summary, this study offers new biomarkers for clinical diagnosis through an in-depth exploration of the mechanisms of action of C1QTNF1-AS1 and miR-34a-5p within osteosarcoma cells, reveals the metabolic characteristics of osteosarcoma, and provides new targets for treatment strategies as well as theoretical support for combination therapy. These findings offer novel insights and approaches for the clinical practice and treatment of osteosarcoma, promising better therapeutic outcomes and quality of life for patients with this condition.

**Figure 8:**
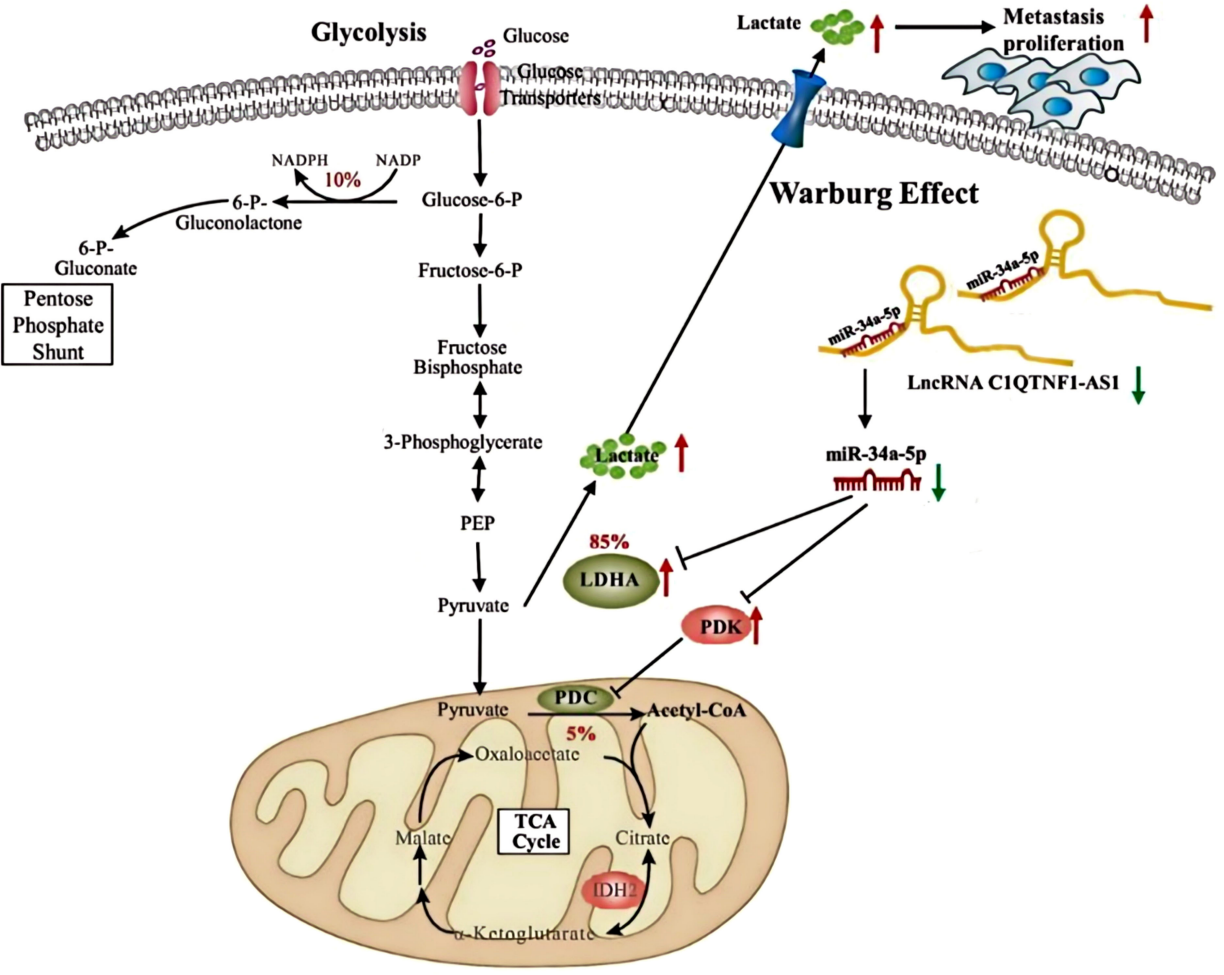
Silent lncRNA-C1QTNF1-AS1 promotes the expression of LDHA and PDK3 by adsorbing miR-34a-5p, indirectly regulating the Warburg effect and facilitating tumor development.

The mechanism diagram of this study can be found in **Figure 8**.

## Data Availability

The datasets presented in this study can be found in online repositories. The names of the repository/repositories and accession number(s) can be found in the article/**Supplementary Material**.
