# Supplementary material for: Deciphering the oncogenic network: how C1QTNF1-AS1 modulates osteosarcoma through miR-34a-5p and glycolytic pathways
Source: Front Oncol. 2025 Jan 9;14:1485605. doi: 10.3389/fonc.2024.1485605 (PMC11754200; doi:10.3389/fonc.2024.1485605)
Supplement: Supplementary file 16 [file Table1.docx]

引物序列

| 引物信息 | 引物序列（5'-3') |
| --- | --- |
| lnc C1QTNF1-AS1 Forward Primer | TCCAAAAGGAGTCTATGGTGAAGTC |
| lnc C1QTNF1-AS1 Reverse Primer | GAAGAAAGATTAGGGGGTCAAAGG |
| miR-34a-5p-Forward Primer | 5’-CACGCATGGCAGTGTCTT-3’ |
| miR-34a-5p-Reverse Primer | 5’-CCAGTGCAGGGTCCGAGGTA-3’ |
| LDHA-Forward Primer | 5’-CAAAGACTACTGTGTAACTGCGA-3’ |
| LDHA-Reverse Primer | 5’-TGGACTGTACTTGACAATGTTGG-3’ |
| PDK3-Forward Primer | 5’-TCCTGGACTTCGGAAGGGATA-3’ |
| PDK3-Reverse Primer | 5’-ACCTCCTCATGGTGTTAGCC-3’ |
| GAPDH-Forward Primer | 5’-AGGTCGGTGTGAACGGATTTG-3’ |
| GAPDH-Reverse Primer | 5’-GGGGTCGTTGATGGCAACA-3’ |
