# Supplementary material for: Deciphering the oncogenic network: how C1QTNF1-AS1 modulates osteosarcoma through miR-34a-5p and glycolytic pathways
Source: Front Oncol. 2025 Jan 9;14:1485605. doi: 10.3389/fonc.2024.1485605 (PMC11754200; doi:10.3389/fonc.2024.1485605)
Supplement: Supplementary file 17 [file Table2.docx]

WB Marker备注：

1. 货号

Prestained Protein Marker II (G2058-250UL) (Servicebio, China)

2. 网址：

https://www.servicebio.cn/goodsdetail?id=6204

3. 文字说明：

选用的Marker条带由上至下为200、140、110、75、55、42、30、23、18、10kDa。

4. 使用抗体：

LDHA Rabbit mAb (A21893)（ABClonal，CHN）稀释比例：1:1000

PDK3 Rabbit pAb (A12480)（ABClonal，CHN）稀释比例：1:1000

β-Actin Rabbit mAb (High Dilution) (AC026)（ABClonal，CHN）稀释比例：1:50000

5. 二抗：

HRP-conjugated Goat anti-Rabbit IgG (H+L) (AS014)（ABClonal，CHN），稀释比1:10000
