# Supplementary material for: Deciphering the oncogenic network: how C1QTNF1-AS1 modulates osteosarcoma through miR-34a-5p and glycolytic pathways
Source: Front Oncol. 2025 Jan 9;14:1485605. doi: 10.3389/fonc.2024.1485605 (PMC11754200; doi:10.3389/fonc.2024.1485605)
Supplement: Supplementary file 19 [file Table4.doc]

abstract

Studies show that osteosarcoma is one of the most common metastatic tumors in children and adolescents, with a poor prognosis. The long noncoding RNA (lncRNA) was found to play an important role as a regulator of cancer proliferation and migration. Among them, lncRNA C1QTNF1-AS1 is an oncogene for a variety of tumors (including colorectal cancer, pancreatic cancer, and hepatocellular carcinoma) and osteosarcoma. This study focused on the function and mechanism of C1QTNF1-AS1 in osteosarcoma. According to bioinformatics analysis, miR-34a-5p is a direct target of C1QTNF1-AS1, and LDHA and PDK 3 are direct targets of miR-34a-5p. The interaction was then verified by a dual-luciferase reporter gene assay. The iments found that C1QTNF1-AS1 expression was significantly decreased in OS cells compared with normal osteoblasts. The expression of miR-34a-5p and C1QTNF1-AS1 showed the same trend in OS cells. Significant enhancement of LDHA and PDK 3 expression in OS cells after silencing C1QTNF1-AS1, while miR-34a-5p mimics partially reversed the promoting effect of LDHA and PDK 3 expression in OS cells after the C1QTNF1-AS1 was rescued. Through functional experiments, we found that silencing of C1QTNF1-AS1 promoted the proliferation, migration, invasive force and warburg effect of OS cells, while miR-34a-5p mimic partially reversed the proliferation, migration, invasive force and warburg effect of cells after silencing of C1QTNF1-AS1. In conclusion, our results clarify that silencing C1QTNF1-AS1 indirectly up-regulates LDHA and PDK 3 expression by inhibiting the expression of miR-34a-5p to regulate Warburg effect and promote tumor development, providing a more powerful rationale for the application of this lncRNA in osteosarcoma.

Key words: osteosarcoma, C1QTNF1-AS1, miR-34a-5p, LDHA, PDK 3, Warburg Effect.

abbreviation

miR : miR-34a-5p

Lnc:LncRNA C1QTNF1-AS1

OS: osteosarcoma

S i: knock down

And mim: overexpression

1. Introduction

Osteosarcoma is a kind of malignant connective tissue tumor in which tumor cells can directly produce tumor bone and osteoid tissue.^1^It is also the most common primary malignant bone tumor, in the incidence of primary malignant tumors.^2^Due to the high degree of malignancy and metastasis, it is difficult to treat. Even after aggressive treatment, the patient's prognosis remains poor.^3,4^The exact etiology of osteosarcoma is still unknown, therefore, it is necessary to reveal the molecular mechanism of osteosarcoma development, identify new molecular markers for early diagnosis, and study new methods for the treatment of this disease.

The Warburg effect means that under hypoxia, cells tend to produce energy via the metabolic pathway of lactate fermentation rather than ATP by oxidative phosphorylation^5^. As a characteristic marker of tumor cells, the main characteristics are active glycolysis, increased glucose consumption, elevated lactate production, and decreased oxygen consumption.^6,7^This extensive study confirms the existence of a Warburg effect in malignant tumor cells and has an important influence on tumor development and progression.^8^

PDK 3 is an enzyme, whose main effect is to affect the energy metabolism of tumor cells by inhibiting the activity of pyruvate dehydrogenase (PDH),^9^Both and the warburg effect are closely related to the energy metabolism and viability of tumor cells. LDHA, a gene related to the Warburg effect and an enzyme related to the glycolytic pathway, promotes the production and accumulation of lactate, thereby maintaining cell survival and proliferation.^10,11^This is one of the main ways that cells can access energy, and according to a previous study this effect is also a feature of OS.

lncRNA (Long non-coding RNA) is a class of non-coding RNA molecules of more than 200 nucleotides in length that do not have the ability to encode proteins and is widely distributed in the human genome.^12^In recent years, researchers have found that lncRNA plays an important promoting or inhibiting role in the formation, occurrence and development of tumors.^13,14^For example, FEZF 1-AS1 regulates the NUPR1-axis by binding to miRNA-4443 to promote OS development.^15^In breast cancer, high expression of C1QTNF1-AS1 was found to affect the proliferation, invasion and metastasis ability of breast cancer cells by regulating multiple signaling pathways, such as Wnt / β -catenin, PI3K / Akt and NF- κ B.^16–18^In lung cancer, high expression of C1QTNF1-AS1 affects key processes such as lung cancer cell proliferation, invasion, and angiogenesis through the regulation of target genes, such as EGFR, HIF-1 α, and VE GF.^19–22^However, the biological function of C1QTNF1-AS1 in OS remains unclear.

At about 22 nucleotides in length, miRNA is a single-stranded non-coding RNA widely studied in recent years.^23^It was found that miR-323a-3p promoted LDHA expression by targeting LDHA could lead to lactate formation and promote metastasis and invasion of head and neck squamous cell cancer cells^24^. The miR-379 was able to inhibit osteosarcoma growth by targeting PDK 1.^25^Several studies have found that miR34a-5p is aberrantly expressed in osteosarcoma cells, and its expression level is closely associated with cell sensitivity, tumor stage, lung metastasis, as well as prognosis.^26,27^However, whether miR-34a-5p is involved in glycolysis and energy metabolism in OS remains unclear.

In this study, we found that the expression of C1QTNF1-AS 1 and miR34a-5p was reduced in OS cells, and that the knockdown of C1QTNF1-AS1 promoted the growth and Warburg effect in OS cells. Moreover, through bioinformatics analysis, C1QTNF1-AS1 has some complementary pairing with miR34a-5p, and LDHA and PDK 3 are potential target genes of miR34a-5p. The interaction was also verified by a dual-luciferase reporter gene assay. We aimed to explore the silence of C1QTNF1-AS1 to promote the expression of target genes such as LDHA catalyzing pyruvate and PDK 3 inhibiting aerobic oxidation of pyruvate by binding to miR-34a-5p, to regulate Warburg effect and promote tumor development and development.

2. Materials and methods

2.1 Cell culture and reagents

Human osteosarcoma cell lines (MG63, Saos 2, U20S, and HOS) and normal human osteoblasts hFOB 1.19 cells were obtained from the Cell Bank of the Chinese Academy of Sciences. Cells were cultured in Eagle medium (containing 10% FBS 0.1%, penicillin, and 0.1% streptomycin). MG63, Saos 2, U20S and HOS cells were incubated with 5%CO2 in humid atmosphere, whereas hFOB 1.19 cells were incubated at 35°C in the same atmosphere.

2.2 This analysis was determined by the RT-qPCR assay

Total RNA extraction was performed using TRIzol (Thermo Fisher Scientific) in OS cells (MG63, SaOS 2, U20S, HOS) according to the supplier introduction. RNA samples were reverse transcribed to cDNA using the PrimeScript RT kit (Takara). RT-qPCR analysis of gene expression was then performed on a ABI7500 Quantitative PCR instrument (ABI Corporation) using SYBR Prex Ex Taq II Kit (Takara) with GAPDH as an internal reference.

2.3 Protein blot

We used a RIPA buffer (Sigma) containing a protease inhibitor (Roche) to extract the total protein. Protein samples were electrophoresed on a 12% SDS-PAGE gel. After transfer, the membranes were blocked with 5% skim milk for 1 h and then incubated overnight with the primary antibody at 4°C. After washing in TBST, the membranes were incubated in the secondary antibodies. Subsequently, the membrane was washed again in TBST and the luminescence was measured using an ECL detection kit (Share-bio). Densitometry analysis of protein-blotted proteins was performed using the ImageJ software.

2.4 Cell Count Kit-8 (CCK-8) test

After transfection, U2OS and MG63 cells were seeded into 96-well plates at a density of 3,103 cells / well. CCK-8 reagent (10 μ L, Dojindo Molecular Technologies) was added to each well and incubated at 37°C and 5%CO 2 for 0 h, 24 and 48 hours. The optical density (OD) at 450nm was detected using a microplate reader (BioTek). Results are representative of three independent experiments.

2.5 cells were transfected

Knodown C1QTNF1-AS1 (si-lnc) and its control si-NC. The miR-34a-5p mimic (miR-mim) and its control (miR-NC) were purchased from GenePharma (Shanghai, China). Transfection was performed using the Lipofectamine 3000 reagent (Invitrogen) according to the manual.

2.6 Measurement of glucose metabolism

Glucose metabolism was measured using a glucose uptake colorimetric test kit (Sigma) according to the method provided in the instructions.

2.7 ATP levels of cells

Cell ATP levels were measured using an ATP assay kit (Promega, Madison, WI, USA) according to the manufacturer's instructions. A fluorescence photometer (Perkin Elmer, Waltham, MA, USA) was used to determine the bioluminescence. ATP levels were calculated from a standard curve.

2.8 Transwell

A matrix-coated 24-well span chamber (8 μ m aperture) was prepared for cell invasion assays. U2OS and MG63 cells in the serum-free medium were incubated in the upper chamber. The medium containing 10% FBS was then added to the lower chamber. After 48 h incubation, cells invading the lower chamber were fixed with methanol and stained with 0.1% crystal violet. Invading cells were observed under an Olympus inverted microscope.

2.9 Cell scratch assay

Migrbility of OS cell lines was assessed using cell scratch assay. When U2OS and MG63 cells reached 90% confluence in 24-well plates, the monolayer was scraped using a sterile plastic tip and washed twice with PBS to remove cell debris. After that, the cells were incubated in a complete growth medium. Finally, cells that migrated to the injured area were collected 0 and 24 hours after the first scratch under an inverted microscope (Olympus) for each wound. Relative distances of cell scratches were analyzed using ImageJ software.

2.10 dual luciferase activity measurement

The Luciferase reporter was performed by cloning wild-type lncRNA-C1QTNF1-AS1 / LDHA / PDK 3 or mutant lncRNA-C1QTNF1-AS1 / LDHA / PDK 3 into the pmirGlo vector (Universal Biol, China). MG63 cells were seeded into 48-well plates at a density of 5,104 cells / well. Luciferase reporter plasmid and miR-34a-5p mim ic / mimic-NC were transfected using liposome 2000 (Invitrogen) for 48 hours. The Dual Luciferase reporter assay system (Promega, USA) was used to assess the activity of the firefly and renilla luciferase enzymes. Firefly luciferase activity was normalized by Renilla luciferase. Each experiment was repeated in three independent experiments.

2.11 Bioinformatics analysis

From the GSE42352 dataset directly from GEO (http: / / www.ncbi.nlm.nih. The gov / geo /) download, consisting of 84 disease samples and 3 healthy controls. In the SangerBox (http: / / sangerbox. Two sets of samples were analyzed by limma package in com /).

2.12 Database analysis

Data on potential miR-34a-5p-target genes were obtained from the online software T arBase (https: / / dianalab.e-ce.uth. Gr /) and miRDB (http: / / mirdb. Og) and TargetScan (http: / / www.targetscan.org/）。According to the RNAhybrid and miRanda website, miR-34a-5p is the target of C1QTNF1-AS1. LDHA, PDK 3 were predicted to be targets of miR-34a-5p based to the TargetScan, miRanda and miRWalk sites.

2.13 Statistical analysis

Data were analyzed and graphed using Graphpad Prism 9 (Version 9.4.0). All data were expressed as means ± SD, and statistical differences between groups were T-Test, with P-value less than 0.05 considered significant.

3. Results

3.1 C1QTNF1-AS1 expression was significantly downregulated in OS cells

To identify differentially expressed genes in OS cells, gene expression profiles GSE42352 used SangerBox to analyze the database in the GEO database. Differentially expressed genes between OS cells and normal osteoblasts were identified using the R package limma (logFC> 1 and p-value <0.05) as indicated in the heatmap and volcano plots (Figure 1A and B). Among these differentially expressed genes, we found that C1QTNF1-AS1 was significantly downregulated in OS samples as compared to normal osteoblasts (Figure 1C). To further confirm the expression of C1QTNF1-AS1 in osteosarcoma cells, we first determined the expression level of C1QTNF1-AS1 in osteoblasts (hFOB 1.19) cells and osteosarcoma cell lines (Saos 2, MG63, HOS, U2OS) by qRT-PCR. The results showed that the expression level of C1QTNF1-AS1 was significantly downregulated in OS cell lines compared with normal human osteogenic cell lines, especially in MG63 and U2OS cell lines.(graph 1D)

3.2 Silencing of C1QTNF1-AS1 significantly promoted the development of OS cells and the warburg effect in vitro

To explore the exact function of C1QTNF1-AS1 in osteosarcoma cells, we established a C1QTNF1-AS1-knockdown cell line (si-lnc) and a negative control (si-NC). According to CCK-8 proliferation assay (Figure 2A), cell scratch assay (Figure 2B) and Transwell assay (Figure 2C), the results showed that C1QTNF1-AS1 promoted the proliferation, migration and invasion of MG63 and U2OS cells. It is well known that the Warburg effect plays a crucial role in tumor development and contributes to the growth of cancer cells.^28^We explored whether C1QTNF1-AS1 is related to the Warburg effect in the development of osteosarcoma by examining the glucose content and the amount of ATP generated in the supernatants of OS cell lines. We found that knockdown of C1QTNF1-AS1 significantly increased the amount of ATP generated in M G63 and U2OS cells, (Figure 2D) but significantly reduced M G63 and U2OS cells, and the glucose content in the supernatant (Figure 2E) (indicating that OS cells consumed more glucose), thus promoting Warburg effector energy metabolism. Taken together, these data suggest that silencing C1QTNF1-AS1 promotes aerobic glycolysis in OS cells.

3.3 miR-34a-5p was identified as a direct target of C1QTNF1-AS1 and showed consistent expression trends in OS cells

To explore the potential mechanism of C1QTNF1-AS1 regulating aerobic glycolysis in OS, we used three target gene prediction algorithms TarBase, miRDB, TargetScan to screen C1QTNF1-AS1-targeted mRNA candidates, and selecting the intersection of three databases revealed that only miR-34a-5p was associated with aerobic glycolysis.(Figure 3A) The binding site of C1QTNF1-AS1 to miR-34a-5p was predicted according to the RNAhybrid and miRanda website, and the mutation sequence mut was designed. The dual-luciferase results showed that miR-34a-5p mimic can bind to C1QTNF1-AS1 wild-type to increase the luciferase activity compared with the expression of mimic NC. However, when the binding site was mutated, miR-34a-5p mimic had no significant effect on the luciferase activity.(Figure 3B) illustrates that miR-34a-5p binds to C1QTNF1-AS1 through this site.(Figure 3C) To further explore the relationship between C1QTNF1-AS1 and miR-34a-5p in OS cells, we established a stable cell line (si-lnc) and a negative control (si-NC), showing by RT-PCR experiments that the expression of miR-34a-5p in OS cells was also relatively reduced after the C1QTNF1-AS1 silencing.(graph 3D)

3.4 miR-34a-5p inhibited the occurrence and development of OS cells by regulating the Warburg effect

To further explore the role of miR-34a-5p in OS, stable cell lines (miR-mim) and negative control (mim-NC) overexpressing miR-34a-5p were established. As shown by CCK-8 proliferation assay, overexpression of miR-34a-5p (Figure 4A), and Transwell test showed that miR-34a-5p inhibited the invasion of OS cells (Figure 4B). The scratch assay showed that the overexpression of miR-34a-5p inhibited the migration force of OS cells.(Figure 4C) We found that overexpression of miR-34a-5p significantly inhibited the ATP production of M G63 and U2OS cells but significantly increased the glucose content in the cell supernatant (indicating that OS cells consumed less glucose), and (Figure 4D and E) thus inhibited the Warburg effect. In conclusion, these results indicate that miR-34a-5p inhibited the invasion, migration, and proliferative forces as well as aerobic glycolysis of OS cells.

3.5 Silencing of C1QTNF1-AS1 promotes OS progression through miR-34-a-5p-mediated glycolysis

To further verify whether C1QTNF1-AS1 achieves the inhibition of OS cell development and progression by targeting miR-34a-5p. Rescue experiments were performed on the U-2OS and MG63 cells. By comparing the results of CCk 8, Transwell and cell scratch assays, we found that silencing C1QTNF1-AS1 significantly promoted the proliferation, migration and invasion of OS cells. However, miR-34a-5p overexpression partially reversed this promoting effect.(Figure 5A-C) Similarly, miR-34a-5p overexpression partially reversed the effect of silencing C1QTNF1-AS1 on the Warburg effect in OS cells.(Figure 5D and E) Together, these findings together suggest that silencing of C1QTNF1-AS1 promotes the proliferation, migration, and invasion of OS cells through inhibition of miR-34a-5p-mediated glycolysis.

3.6 LDHA, PDK 3 were identified as a direct target of miR-34a-5p and showed opposite trends in OS cells

The binding sites of LDHA, PDK 3 and miR-34a-5p were predicted according to the TargetScan, miRanda and miRWalk sites, and both sites mutated the sequence mut. The dual-luciferase results showed that miR-34a-5p mimic could bind to LDHA and PDK 3 wild-type to reduce the luciferase activity compared with the expression of mimic-NC. However, when the binding site was mutated, miR-34a-5p mimic had no significant effect on the luciferase activity.(Figure 6A and B) illustrates that miR-34a-5p binds to LDHA and PDK 3 through this site (Figure 6C and D). We established stable cell lines overexpressing miR-34a-5p (miR-mim) and a negative control (mim-NC). PDK 3 expression in OS cells was significantly decreased in LDHA after miR-34a-5p as determined by RT-PCR and western blot.(Figure 6 E and F)

3.7 Silencing of C1QTNF1-AS1 upregulates the expression of LDHA, PDK 3 in OS cells through inhibition of miR-34a-5p

To explore the interaction of C1QTNF1-AS1, miR-34a-5p and LDHA and PDK 3 in OS cells, we constructed knockdown C1QTNF1-AS1 stable cell lines (si-lnc) and miR-34a-5p overexpression stable cell lines (miR-mim). Then, by RT-PCR and protein blot, we found that the expression of PDK 3 in OS cells increased after silencing of C1QTNF1-AS1 (Figure 7A and B), while the expression of LDHA and PDK 3 in OS cells decreased after miR-34a-5p overexpression (Figure 7C and D). Through rescue experiments, we found that the overexpression of miR-34a-5p could partially reverse the promoting effect of LDHA and PDK 3 after C1QTNF1-AS1 silencing (Figure 7E and F). Taken together, these results indicate that silencing C1QTNF1-AS1 upregulates LDHA, PDK 3 expression in OS cells through inhibition of miR-34a-5p.

4. Discuss

Osteosarcoma (Osteosarcoma, OS), as a common clinical primary bone tumor, is mostly concentrated in adolescents or children, and its incidence ranks first among primary bone malignant tumors.^29^The tumor is mainly characterized by rapid progression and easy metastasis, and it is a highly malignant bone tumor.^30^Recent studies suggest that lncRNA plays a crucial role in the progression of various cancers.^31,32^In this topic, we focused on the mechanism and function of lncRNA C1QTNF1-AS1 in OS. The results showed that silencing of C1QTNF1-AS1 promoted the expression of LDHA and PDK 3 by adsorption of miR-34a-5p.

Increasing evidence suggests that dysregulation of C1QTNF1-AS1 is associated to many processes of tumor progression, including OS.^21,33^Previous studies have shown that C1QTNF1-AS1 is significantly downregulated in some tumor cells, and that C1QTNF1-AS1 suppresses a variety of cancers (e. g., colorectal, ovarian, pancreatic, gastric and hepatocellular carcinoma.) Proliferation, invasion, epithelial-interstitial transformation (EMT), and induction of apoptosis,^20,32,34,35^C1QTNF1-AS1 has been found to the Warburg effect in colorectal cancer.^36^In this study, we validated the low C1QTNF1-AS1 expression in OS cells. Functional experiments showed that silencing of C1QTNF1-AS1 promoted the proliferation, migration, invasion, and Warburg effects of OS cells. Taken together, C1QTNF1-AS1 plays a crucial role in OS progression.

Several studies have demonstrated that miR-34a-5p can inhibit malignancy in the cervix by inhibiting cell proliferation and invasion.^37^The miR-34a-5p inhibited the development of HNSCC by targeting Flotillin-2.^27^In this study, miR-34a-5p was predicted to be a C1QTNF1-AS1 target, and this interaction was validated by a dual-luciferase reporter gene assay. We experimentally verified a positive correlation between the expression trend of miR-34a-5p and C1QTNF1-OS 1 expression in OS cells. Subsequently, we verified the inhibition of cell proliferation, migration, invasion, and Warburg effects of OS cells after miR-34a-5p overexpression by functional experiments. Furthermore, silencing C1QTNF1-AS1 demonstrated OS cell proliferation, invasion, migration and Warburg effects by adsorbing miR-34a-5p. These data show that C1QTNF1-AS1 regulates OS progression by adsorbing the miR-34a-5p.

Numerous studies have confirmed the existence of a Warburg effect in malignant tumor cells and has an important influence on tumor development and progression.^38^LDHA is also a key enzyme in the last step of the Warburg effect, is highly expressed in many tumor cells, and is closely correlated with tumor size and prognosis.^39,40^PDK has four subtypes (PDK 1, PDK 2, PDK 3, PDK 4), closely related to the generation of Warburg effect, and is central at the crossroads of glycolysis and oxidative phosphorylation.^41,42^In this study, we predicted LDHA and PDK 3 as direct targets of miR-34a-5p, followed by dual-luciferase reporter gene assay. Moreover, the expression of C1QTNF1-AS1, miR-34a-5p and LDHA and PDK 3 in OS cells, while rescue experiments showed that silencing C1QTNF1-AS1 upregulated LDHA and PDK 3 expression levels in OS cells by adsorption of miR-34a-5p.

In conclusion, silencing lncRNA C1QTNF1-AS1 regulates the expression of target genes by adsorbing miR-34a-5p to promote LDHA and PDK 3 of pyruvate, and promote the development and development of osteosarcoma.

reference documentation

(1) Corre, I.; Verrecchia, F.; Crenn, V.; Redini, F.; Trichet, V.The Osteosarcoma Microenvironment: A Complex but Targetable Ecosystem.Cells 2020, 9 (4), 976. https://doi.org/10.3390/cells9040976.

(2) Shen, Y.; Xu, J.; Pan, X.; Zhang, Y.; Weng, Y.; Zhou, D.; He, S.LncRNA KCNQ1OT1 Sponges miR-34c-5p to Promote Osteosarcoma Growth via ALDOA Enhanced Aerobic Glycolysis.Cell Death Dis.2020, 11 (4), 278. https://doi.org/10.1038/s41419-020-2485-1.

(3) Pan, X.; Li, H.; Tan, J.; Weng, X.; Zhou, L.; Weng, Y.; Cao, X.miR-1297 Suppresses Osteosarcoma Proliferation and Aerobic Glycolysis by Regulating PFKFB2. OncoTargets and therapy 2020, 13, 11265–11275. https://doi.org/10.2147/OTT.S274744.

(4) Lu, J.; Song, G.; Tang, Q.; Zou, C.; Han, F.; Zhao, Z.; Yong, B.; Yin, J.; Xu, H.; Xie, X.; Kang, T.; Lam, Y.; Yang, H.; Shen, J.; Wang, J.IRX1 Hypomethylation Promotes Osteosarcoma Metastasis via Induction of CXCL14/NF-κB Signaling.J.Clin.Invest.2015, 125 (5), 1839–1856. https://doi.org/10.1172/JCI78437.

(5) Weng, Y.; Duan, W.; Yu, X.; Wu, F.; Yang, D.; Jiang, Y.; Wu, J.; Wang, M.; Wang, X.; Shen, Y.; Zhang, Y.; Xu, H.MicroRNA‐324‐3p Inhibits Osteosarcoma Progression by Suppressing PGAM1‐mediated Aerobic Glycolysis.Cancer Sci.2023, 114 (6), 2345–2359. https://doi.org/10.1111/cas.15779.

(6) Chelakkot, C.; Chelakkot, V.S.; Shin, Y.; Song, K.Modulating Glycolysis to Improve Cancer Therapy.International Journal of Molecular Sciences 2023, 24 (3), 2606. https://doi.org/10.3390/ijms24032606.

(7) FOXO3A-induced LINC00926 suppresses breast tumor growth and metastasis through inhibition of PGK 1-mediated Warburg effect-PMC.https://www.ncbi.nlm.nih.gov/pmc/articles/PMC8417517/ (accessed 2024-07-06).

(8) Zhong, X.; He, X.; Wang, Y.; Hu, Z.; Huang, H.; Zhao, S.; Wei, P.; Li, D.Warburg Effect in Colorectal Cancer: The Emerging Roles in Tumor Microenvironment and Therapeutic Implications.J.Hematol.Oncol.2022, 15, 160. https://doi.org/10.1186/s13045-022-01358-5.

(9) Atas, E.; Oberhuber, M.; Kenner, L.The Implications of PDK1–4 on Tumor Energy Metabolism, Aggressiveness and Therapy Resistance.Frontiers in Oncology 2020, 10. https://doi.org/10.3389/fonc.2020.583217.

(10) Pathria, G.; Scott, D.A.; Feng, Y.; Sang Lee, J.; Fujita, Y.; Zhang, G.; Sahu, A.D.; Ruppin, E.; Herlyn, M.; Osterman, A.L.; Ronai, Z.A.Targeting the Warburg Effect via LDHA Inhibition Engages ATF4 Signaling for Cancer Cell Survival.Embo J.2018, 37 (20), e99735. https://doi.org/10.15252/embj.201899735.

(11) Xu, K.; Yin, N.; Peng, M.; Stamatiades, E.G.; Shyu, A.; Li, P.; Zhang, X.; Do, M.H.; Wang, Z.; Capistrano, K.J.; Chou, C.; Levine, A.G.; Rudensky, A.Y.; Li, M.O.Glycolysis Fuels Phosphoinositide 3-Kinase Signaling to Bolster T Cell Immunity.Science (New York, N.Y.) 2021, 371 (6527), 405–410. https://doi.org/10.1126/science.abb2683.

(12) Yang, M.; Zheng, H.; Xu, K.; Yuan, Q.; Aihaiti, Y.; Cai, Y.; Xu, P.A Novel Signature to Guide Osteosarcoma Prognosis and Immune Microenvironment: Cuproptosis-Related lncRNA.Front.Immunol.2022, 13, 919231. https://doi.org/10.3389/fimmu.2022.919231.

(13) Xing, C.; Sun, S.; Yue, Z.-Q.; Bai, F.Role of lncRNA LUCAT1 in Cancer.Biomedicine & Pharmacotherapy 2021, 134, 111158. https://doi.org/10.1016/j.biopha.2020.111158.

(14) Chi, Y.; Wang, D.; Wang, J.; Yu, W.; Yang, J.Long Non-Coding RNA in the Pathogenesis of Cancers.Cells 2019, 8 (9), 1015. https://doi.org/10.3390/cells8091015.

(15) Liu, J.; Feng, G.; Li, Z.; Li, R.; Xia, P.Long Non-Coding RNA FEZF1-AS1 Modulates CXCR4 to Promote Cell Proliferation, Warburg Effect and Suppress Cell Apoptosis in Osteosarcoma by Sponging miR-144. OncoTargets and therapy 2020, 13, 2899–2910. https://doi.org/10.2147/OTT.S235970.

(16) Pu, F.; Liu, J.; Jing, D.; Chen, F.; Huang, X.; Shi, D.; Wu, W.; Lin, H.; Zhao, L.; Zhang, Z.; Lv, X.; Wang, B.; Zhang, Z.; Shao, Z.LncCCAT1 Interaction Protein PKM2 Upregulates SREBP2 Phosphorylation to Promote Osteosarcoma Tumorigenesis by Enhancing the Warburg Effect and Lipogenesis.Int.J.Oncol.2022, 60 (4), 44. https://doi.org/10.3892/ijo.2022.5334.

(17) Hajibabaei, S.; Nafissi, N.; Azimi, Y.; Mahdian, R.; Rahimi-Jamnani, F.; Valizadeh, V.; Rafiee, M.H.; Azizi, M.Targeting Long Non-Coding RNA MALAT1 Reverses Cancerous Phenotypes of Breast Cancer Cells through microRNA-561-3p/TOP2A Axis.Sci.Rep.2023, 13, 8652. https://doi.org/10.1038/s41598-023-35639-x.

(18) Xiu, B.; Chi, Y.; Liu, L.; Chi, W.; Zhang, Q.; Chen, J.; Guo, R.; Si, J.; Li, L.; Xue, J.; Shao, Z.-M.; Wu, Z.-H.; Huang, S.; Wu, J.LINC02273 Drives Breast Cancer Metastasis by Epigenetically Increasing AGR2 Transcription.Mol Cancer 2019, 18, 187. https://doi.org/10.1186/s12943-019-1115-y.

(19) Shen, Y.; Hou, N.; Han, F.; Chen, B.; Shi, J.; Sun, X.Comprehensive Analysis of Tumor Immune Microenvironment and Prognosis of m6A-Related IncRNAs in Lung Adenocarcinoma.Crit.Rev.Eukaryot.Gene Expr.2022, 32 (5), 77–91. https://doi.org/10.1615/CritRevEukaryotGeneExpr.2022042417.

(20) Schwerdtfeger, M.; Desiderio, V.; Kobold, S.; Regad, T.; Zappavigna, S.; Caraglia, M.Long Non-Coding RNAs in Cancer Stem Cells.Transl.Oncol.2021, 14 (8), 101134. https://doi.org/10.1016/j.tranon.2021.101134.

(21) Han, W.; Yu, G.; Meng, X.; Hong, H.; Zheng, L.; Wu, X.; Zhang, D.; Yan, B.; Ma, Y.; Li, X.; Wang, Q.Potential of C1QTNF1-AS1 Regulation in Human Hepatocellular Carcinoma.Mol.Cell.Biochem.2019, 460 (1), 37–51. https://doi.org/10.1007/s11010-019-03569-w.

(22) Hua, Q.; Jin, M.; Mi, B.; Xu, F.; Li, T.; Zhao, L.; Liu, J.; Huang, G.LINC01123, a c-Myc-Activated Long Non-Coding RNA, Promotes Proliferation and Aerobic Glycolysis of Non-Small Cell Lung Cancer through miR-199a-5p/c-Myc Axis.J Hematol Oncol 2019, 12, 91. https://doi.org/10.1186/s13045-019-0773-y.

(23) Kousar, K.; Ahmad, T.; Abduh, M.S.; Kanwal, B.; Shah, S.S.; Naseer, F.; Anjum, S.miRNAs in Regulation of Tumor Microenvironment, Chemotherapy Resistance, Immunotherapy Modulation and miRNA Therapeutics in Cancer.International Journal of Molecular Sciences 2022, 23 (22), 13822. https://doi.org/10.3390/ijms232213822.

(24) Celik, B.; Cicek, K.; Leal, A.F.; Tomatsu, S.Regulation of Molecular Targets in Osteosarcoma Treatment.International Journal of Molecular Sciences 2022, 23 (20), 12583. https://doi.org/10.3390/ijms232012583.

(25) Jiang, M.; Jike, Y.; Liu, K.; Gan, F.; Zhang, K.; Xie, M.; Zhang, J.; Chen, C.; Zou, X.; Jiang, X.; Dai, Y.; Chen, W.; Qiu, Y.; Bo, Z.Exosome-Mediated miR-144-3p Promotes Ferroptosis to Inhibit Osteosarcoma Proliferation, Migration, and Invasion through Regulating ZEB1. Mol.Cancer 2023, 22, 113. https://doi.org/10.1186/s12943-023-01804-z.

(26) Pu, Y.; Zhao, F.; Wang, H.; Cai, S.MiR-34a-5p Promotes Multi-Chemoresistance of Osteosarcoma through down-Regulation of the DLL1 Gene.Sci.Rep.2017, 7, 44218. https://doi.org/10.1038/srep44218.

(27) Li, X.; Zhao, S.; Fu, Y.; Zhang, P.; Zhang, Z.; Cheng, J.; Liu, L.; Jiang, H.miR-34a-5p Functions as a Tumor Suppressor in Head and Neck Squamous Cell Cancer Progression by Targeting Flotillin-2. Int J Biol Sci 2021, 17 (15), 4327–4339. https://doi.org/10.7150/ijbs.64851.

(28) Halma, M.T.J.; Tuszynski, J.A.; Marik, P.E.Cancer Metabolism as a Therapeutic Target and Review of Interventions.Nutrients 2023, 15 (19), 4245. https://doi.org/10.3390/nu15194245.

(29) Bian, J.; Liu, Y.; Zhao, X.; Meng, C.; Zhang, Y.; Duan, Y.; Wang, G.Research Progress in the Mechanism and Treatment of Osteosarcoma.Chin Med J (Engl) 2023, 136 (20), 2412–2420. https://doi.org/10.1097/CM9.0000000000002800.

(30) Shoaib, Z.; Fan, T.M.; Irudayaraj, J.M.K.Osteosarcoma Mechanobiology and Therapeutic Targets.Br J Pharmacol 2022, 179 (2), 201–217. https://doi.org/10.1111/bph.15713.

(31) Yu, X.; Duan, W.; Wu, F.; Yang, D.; Wang, X.; Wu, J.; Zhou, D.; Shen, Y.LncRNA‐HOTAIRM1 Promotes Aerobic Glycolysis and Proliferation in Osteosarcoma via the miR‐664b‐3p/Rheb/mTOR Pathway.Cancer Sci.2023, 114 (9), 3537–3552. https://doi.org/10.1111/cas.15881.

(32) Park, E.-G.; Pyo, S.-J.; Cui, Y.; Yoon, S.-H.; Nam, J.-W.Tumor Immune Microenvironment lncRNAs.Brief.Bioinform.2021, 23 (1), bbab504. https://doi.org/10.1093/bib/bbab504.

(33) Cagle, P.; Qi, Q.; Niture, S.; Kumar, D.KCNQ1OT1: An Oncogenic Long Noncoding RNA.Biomolecules 2021, 11 (11), 1602. https://doi.org/10.3390/biom11111602.

(34) Tan, Y.; Lin, J.; Li, T.; Li, J.; Xu, R.; Ju, H.LncRNA‐mediated Posttranslational Modifications and Reprogramming of Energy Metabolism in Cancer.Cancer Commun.2020, 41 (2), 109–120. https://doi.org/10.1002/cac2.12108.

(35) Huang, Z.; Zhou, J.-K.; Peng, Y.; He, W.; Huang, C.The Role of Long Noncoding RNAs in Hepatocellular Carcinoma.Mol Cancer 2020, 19, 77. https://doi.org/10.1186/s12943-020-01188-4.

(36) Park, M.K.; Zhang, L.; Min, K.-W.; Cho, J.-H.; Yeh, C.-C.; Moon, H.; Hormaechea-Agulla, D.; Mun, H.; Ko, S.; Lee, J.W.; Jathar, S.; Smith, A.S.; Yao, Y.; Giang, N.T.; Vu, H.H.; Yan, V.C.; Bridges, M.C.; Kourtidis, A.; Muller, F.; Chang, J.H.; Song, S.J.; Nakagawa, S.; Hirose, T.; Yoon, J.-H.; Song, M.S.NEAT1 Is Essential for Metabolic Changes That Promote Breast Cancer Growth and Metastasis.Cell Metab 2021, 33 (12), 2380-2397.e9. https://doi.org/10.1016/j.cmet.2021.11.011.

(37) Jiang, T.; Cheng, H.miR-34a-5p Blocks Cervical Cancer Growth and Migration by Downregulating CDC25A.

(38) Stine, Z.E.; Schug, Z.T.; Salvino, J.M.; Dang, C.V.Targeting Cancer Metabolism in the Era of Precision Oncology.Nat.Rev.Drug Discov.2022, 21 (2), 141–162. https://doi.org/10.1038/s41573-021-00339-6.

(39) Xia, P.; Zhang, H.; Lu, H.; Xu, K.; Jiang, X.; Jiang, Y.; Gongye, X.; Chen, Z.; Liu, J.; Chen, X.; Ma, W.; Zhang, Z.; Yuan, Y.METTL5 Stabilizes c‐Myc by Facilitating USP5 Translation to Reprogram Glucose Metabolism and Promote Hepatocellular Carcinoma Progression.Cancer Commun.2023, 43 (3), 338–364. https://doi.org/10.1002/cac2.12403.

(40) Jing, Z.; Liu, Q.; He, X.; Jia, Z.; Xu, Z.; Yang, B.; Liu, P.NCAPD3 Enhances Warburg Effect through C-Myc and E2F1 and Promotes the Occurrence and Progression of Colorectal Cancer.J Exp Clin Cancer Res 2022, 41, 198. https://doi.org/10.1186/s13046-022-02412-3.

(41) Sun, W.-H.; Chen, Y.-H.; Lee, H.-H.; Tang, Y.-W.; Sun, K.-H.PDK1and PDK2-Mediated Metabolic Reprogramming Contributes to the TGFβ1-Promoted Stem-like Properties in Head and Neck Cancer.Cancer Metab 2022, 10, 23. https://doi.org/10.1186/s40170-022-00300-0.

(42) Luengo, A.; Li, Z.; Gui, D.Y.; Sullivan, L.B.; Zagorulya, M.; Do, B.T.; Ferreira, R.; Naamati, A.; Ali, A.; Lewis, C.A.; Thomas, C.J.; Spranger, S.; Matheson, N.J.; Vander Heiden, M.G.Increased Demand for NAD+ Relative to ATP Drives Aerobic Glycolysis.Molecular cell 2021, 81 (4), 691-707.e6. https://doi.org/10.1016/j.molcel.2020.12.012.
