# Supplementary material for: Deciphering the oncogenic network: how C1QTNF1-AS1 modulates osteosarcoma through miR-34a-5p and glycolytic pathways
Source: Front Oncol. 2025 Jan 9;14:1485605. doi: 10.3389/fonc.2024.1485605 (PMC11754200; doi:10.3389/fonc.2024.1485605)
Supplement: Supplementary file 21 [file Table6.doc]

**Mechanism of LncRNA C1QTNF1-AS1 multitarget regulation of LDHA/PDK3 mediated Warburg effect in osteosarcoma**

**Yu Zhang^ab^, Hailong Lun ^c^，Naiqiang Zhu^a*^, Ning Yang^b^, Kaikai Ding^ab^, Jialu Wang^d^ , Bin Chen^a^ , Chengbing Chang^a^, Haipeng Gu^a^ , Yanqi Liu^a^**

^a^ Department of Minimally Invasive Spinal Surgery, The Affiliated Hospital of Chengde Medical University, Chengde, Hebei, China

^b^ Hebei Key Laboratory of Panvascular Diseases, Chengde, Hebei, China

^c^ Tangshan Nanhu Hospital,Tangshan,Hebei,China

^d^ School of Public Health, Hebei Medical University,Shijiazhuang,Hebei,China

**Corresponding Author:**

Naiqiang Zhu*, Department of Minimally Invasive Spinal Surgery, The Affiliated Hospital of Chengde Medical University, No.36 Nanyingzi Street, Chengde, Hebei, 067000, China

Email address: zhunq2010@.163com

**Abstract**

**Background**

Osteosarcoma (OS) is one of the most common metastatic tumors in children and adolescents and has a poor prognosis. Long noncoding RNA (lncRNA) play an important role as regulators of cancer proliferation and migration. Among them, C1QTNF1-AS1 is an oncogene for a variety of tumors (such as colorectal cancer, pancreatic cancer, and hepatocellular carcinoma) including OS. This study focused on the functions and mechanism of lncRNA C1QTNF1-AS1 in osteosarcoma.

**Methods**

In this study, we focused on the function and mechanism of action of C1QTNF1-AS1 in OS. Bioinformatics analysis revealed that microRNA (miR)-34a-5p is a direct target of C1QTNF1-AS1, and LDHA and PDK3 are direct targets of miR-34a-5p. This interaction was verified using the dual-luciferase reporter assay. The expression trend of C1QTNF1-AS1, miR-34a-5p, LDHA, and PDK3 in OS cells were determined by RT-PCR and westernblot, and the targeting relationship between the four subjects. The proliferation, invasion and migration force changes of C1QTNF1-AS1 and miR-34a-5p in OS cells were verified by CCK 8, Transwel, cell scratch assay. The aerobic glycolysis levels of C1QTNF1-AS1 and miR-34a-5p in OS cells were assessed by measuring ATP levels and glucose uptake capacity.

**Result**

The results showed that C1QTNF1-AS1 expression was significantly lower in OS cells than in normal osteoblasts. The expression levels of miR-34a-5p and C1QTNF1-AS1 showed a similar trend in OS cells. Significant enhancement of LDHA and PDK3 expression in OS cells was observed after silencing C1QTNF1-AS1, whereas miR-34a-5p mimics partially reversed the promoting effect of LDHA and PDK3 expression in OS cells . Functional experiments revealed that silencing C1QTNF1-AS1 promoted the proliferation, migration, invasive force, and Warburg effect in OS cells, whereas the miR-34a-5p mimic partially reversed these proliferation,migration, invasive force, and Warburg effects.

**Conclude**

In conclusion, our results indicate that silencing C1QTNF1-AS1 indirectly upregulates LDHA and PDK3 expression by inhibiting the expression of miR-34a-5p, which regulates the Warburg effect, and thereby promotes OS development, providing a powerful rationale for the application of this lncRNA in OS.

**Keywords**: OS, C1QTNF1-AS1, miR-34a-5p, LDHA, PDK3, Warburg Effect.

**1. Background**

Osteosarcoma (OS) is a type of malignant connective tissue tumor in which tumor cells can directly produce tumor bone and osteoid tissue.^1^ It is also the most common primary malignant bone tumor among primary malignant tumors.^2^ Treatment is difficult owing to the high degree of malignancy and metastasis. Even after aggressive treatment, the prognosis of patients remains poor.^3,4^ The exact etiology of OS remains unknown; therefore, it is necessary to reveal the molecular mechanism of OS development, identify new molecular markers for early diagnosis, and study new methods for the treatment of this disease.

The Warburg effect implies that under hypoxia, cells tend to produce energy via the lactate fermentation metabolic pathway rather than using ATP via oxidative phosphorylation.^5^ The main characteristics of tumor cells are active glycolysis, increased glucose consumption, elevated lactate production, and decreased oxygen consumption.^6^ The existence of the Warburg effect in malignant tumor cells, which has an important influence on tumor development and progression, has been confirmed through extensive study.^7^

Pyruvate Dehydrogenase Kinase 3 (PDK3) is a protein enzyme involved in the regulation of intracellular energy metabolism. PDK3 is an enzyme that mainly affects the energy metabolism of tumor cells by inhibiting the activity of pyruvate dehydrogenase.^8^ The Warburg effect is also closely related to energy metabolism and tumor cell viability. Lactate Dehydrogenase A (LDHA) is an enzyme that is involved in the intracellular lactate metabolism. LDHA, as a gene related to the Warburg effect and an enzyme related to the glycolytic pathway, promotes the production and accumulation of lactate, thereby maintaining cell survival and proliferation.^9,10^ This is one of the main pathways by which cells can access energy, and according to a previous study, ^11^this effect is also a feature of OS.

Long noncoding RNAs (lncRNAs) are a class of noncoding RNA molecules more than 200 nucleotides in length that do not have the ability to encode proteins and are widely distributed in the human genome.^12^ Studies have reported lncRNA as playing an important promoting or inhibiting role in the formation, occurrence, and development of tumors.^13,14^ For example, FEZF1-AS1 regulates the NUPR1-axis by binding to microRNA (miRNA)-4443 to promote OS development.^15^ In breast cancer, high expression of C1QTNF1-AS1 was found to affect the proliferation, invasion, and metastasis ability of breast cancer cells by regulating multiple signaling pathways, such as the Wnt/β-catenin, PI3K/Akt, and NF-κB pathways.^16–18^ In lung cancer, high expression of C1QTNF1-AS1 affects key processes such as cell proliferation, invasion, and angiogenesis through the regulation of target genes including EGFR, HIF-1 α, and VEGF.^19–22^ However, the biological function of C1QTNF1-AS1 in OS remains unclear.

At approximately 22 nucleotides in length, miRNA is a single-stranded noncoding RNA that has been widely studied.^23^ Promotion of LDHA expression by targeting of LDHA by miR-323a-3p led to lactate formation and promotion of metastasis and invasion of head and neck squamous cell cancer cells.^24^ Osteosarcoma growth was inhibited by miR-379 by targeting PDK1.^25^ Several studies have found that miR34a-5p is aberrantly expressed in OS cells, and its expression level is closely associated with cell sensitivity, tumor stage, lung metastasis, as well as prognosis.^26,27^ However, whether miR-34a-5p is involved in glycolysis and energy metabolism in OS remains unclear.

In the present study, we found that the expression of C1QTNF1-AS1 and miR-34a-5p was reduced in OS cells, and that C1QTNF1-AS1 knockdown promoted growth and the Warburg effect in OS cells. Moreover, bioinformatics analysis showed that C1QTNF1-AS1 has some complementary pairing with miR-34a-5p, and LDHA and PDK3 are potential target genes of miR-34a-5p. This interaction was verified using the dual-luciferase reporter assay. We aimed to explore that silencing C1QTNF1-AS1 indirectly promoted the expression of LDHA and PDK3 by inhibiting the expression of miR-34a-5p, thus regulating Warburg effect and promoting tumor development and development.

**2. Methods**

**2.1 Cell culture and reagents**

Human OS cell lines (MG63, Saos-2, U20S, and HOS) and normal human osteoblasts (hFOB 1.19) were obtained from the Cell Bank of the American Type Culture Collection (ATCC, Manassas, VA, USA).All cells were cultured in Eagle’s medium containing 10% Fetal Bovine Serum (Thermo Fisher Scientific), 0.1% penicillin, and 0.1% streptomycin((Invitrogen, Carlsbad, CA, USA). in an incubator with the parameters of 37°C and 5% CO_2_

**2**.**2 Quantitative Real-Time Polymerase Chain Reaction(qRT-PCR)**

Total RNA was extracted from OS cells (MG63, Saos-2, U20S, and HOS) using TRIzol reagent (Thermo Fisher Scientific) according to the manufacturer’s instructions. RNA samples were reverse transcribed to cDNA using the PrimeScript RT Kit (Takara). Gene expression was analyzed using RT-qPCR performed on an ABI7500 Quantitative PCR instrument (ABI Corporation) using an SYBR Prex Ex Taq II Kit (Takara), with GAPDH as an internal reference.

**2**.**3 Western blotting**

RIPA buffer (Sigma) containing a protease inhibitor (Roche) was used to extract the total protein. Protein samples were electrophoresed on a 12% sodium dodecyl sulfate-polyacrylamide gel. After transfer, the membranes were blocked with 5% skim milk for 1 h and then incubated overnight with the primary antibody at 4°C. After washing with Tris-Buffered Saline with Tween（TBST）, the membranes were incubated with secondary antibodies. Subsequently, the membranes were washed again in TBST and luminescence was measured using an ECL detection kit (Share-bio). Densitometric analysis of the blotted proteins was performed using ImageJ software

.

**2.4 Cell Count Kit-8 (CCK-8) test**

After transfection, U2OS and MG63 cells were seeded in 96-well plates at a density of 3×10^3^ cells/well. The CCK-8 reagent (10 μL, Dojindo Molecular Technologies) was added to each well and incubated at 37°C and 5% CO_2_ for 0, 24, and 48 h. The optical density (OD) was measured at 450 nm using a microplate reader (BioTek). The results are presented as representatives of three independent experiments.

**2.5 Cell transfection**

The C1QTNF1-AS1 knockdown (si-lnc) and a negative control (si-NC) were constructed. An miR-34a-5p analog (miR-mim) and its control (miR-NC) were purchased from GenePharma (Shanghai, China). Transfection was performed using the Lipofectamine 3000 reagent (Invitrogen) according to the manufacturer’s instructions.

**2.6 Measurement of** **glucose metabolism and the cell ATP levels**

Glucose metabolism was measured using a glucose uptake colorimetric test kit (Sigma) according to the manufacturer’s instructions. Cellular ATP levels were measured using an ATP assay kit (Promega, Madison, WI, USA) according to the manufacturer's instructions. A fluorescence photometer (Perkin Elmer, Waltham, MA, USA) was used to measure bioluminescence. The ATP levels were calculated using a standard curve.

**2.7 Transwell assay**

A matrix-coated 24-well span chamber (8 μm aperture) was prepared for cell invasion assays. U2OS and MG63 cells were incubated in serum-free medium in the upper chamber. Medium containing 10% FBS was added to the lower chamber. After 48 h of incubation, the cells invading the lower chamber were fixed with methanol and stained with 0.1% crystal violet. The invading cells were observed under an inverted microscope (Olympus).

**2.8 Cell scratch assay**

Migration of OS cell lines was assessed using a cell scratch assay. When the U2OS and MG63 cells reached 90% confluence in 24-well plates, the monolayer was scraped using a sterile plastic tip and washed twice with Phosphate-Buffered Saline （PBS） to remove cell debris. The cells were then incubated in a complete growth medium. Finally, cells that migrated to the injured area were collected at 0 and 24 h after the first scratch and observed under an inverted microscope (Olympus) for each wound. The relative distances of the cell scratches were analyzed using the ImageJ software.

**2.9 Dual luciferase activity measurement**

The dual luciferase reporter assay was performed by cloning wild-type lncRNA-C1QTNF1-AS1/LDHA/PDK3 or mutant lncRNA-C1QTNF1-AS1/LDHA/PDK3 into the pmirGLO vector (Universal Biotech , China). MG63 cells were seeded in 48-well plates at a density of 5×10^4^ cells/well. Luciferase reporter plasmid and miR-34a-5p mimic/mimic-NC were transfected for 48 h using Liposome 2000 (Invitrogen). A dual luciferase reporter assay system (Promega, USA) was used to assess the activity of firefly and Renilla luciferase enzymes. Firefly luciferase activity was normalized to that of the Renilla luciferase. Each experiment was repeated three times.

**2.10 Bioinformatics analysis**

The GSE42352 dataset was obtained from the Gene Expression Omnibus (http://www.ncbi.nlm.nih.gov/geo). The downloaded data consisted of 84 disease samples and three healthy controls. Two sample sets were analyzed using the SangerBox platform (http://sangerbox.com) and the limma package（Linear Models for Microarray Data version **3.46.0** from Bioconductor）.

**2.11. Database analysis**

Data on potential miR-34a-5p-target genes were obtained using the online software packages TarBase (https://dianalab.e-ce.uth.gr), miRDB (https://mirdb.org), and TargetScan (https://[www.targetscan.org/](http://www.targetscan.org/)). According to the RNAhybrid and miRanda websites, miR-34a-5p is a target of C1QTNF1-AS1. Based on the TargetScan, miRanda, and miRWalk prediction databases, LDHA and PDK3 were predicted to be targets of miR-34a-5p.

**2.12 Statistical analysis**

Data were analyzed and graphed using GraphPad Prism 9 (version 9.4.0). All data are expressed as mean ± SD, and statistical differences between groups were determined using the *t*-test. Statistical significance was set at *p* < 0.05.

**3.** **Results**

**3.1 C1QTNF1-AS1 expression was significantly downregulated in OS cells**

To identify differentially expressed genes in OS cells, gene expression profiles in the GSE42352 dataset obtained from GEO were analyzed using the SangerBox platform. Differentially expressed genes between OS cells and normal osteoblasts were identified using the limma package (log FC > 1 and *p* < 0.05) and the heatmap and volcano plots prepared in The R Project for Statistical Computing（R version 4.4.0）(Fig.1A andB). Among these differentially expressed genes, we found that C1QTNF1-AS1 was significantly downregulated in OS cells compared to that in normal osteoblasts (Fig.1C). We first determined the expression levels of C1QTNF1-AS1 in osteoblasts (hFOB 1.19) and osteosarcoma cell lines (Saos-2, MG63, HOS, and U2OS) using qRT-PCR. The results showed that the expression of C1QTNF1-AS1 was significantly downregulated in OS cell lines compared to that in normal human osteogenic cell lines, especially in MG63 and U2OS cell lines (Fig.1D).

**3.2** **Silencing of C1QTNF1-AS1 significantly promoted OS cell development and the Warburg effect in vitro**

To explore the exact function of C1QTNF1-AS1 in OS cells, we established a C1QTNF1-AS1-knockdown cell line (si-lnc) and a negative control (si-NC). Results of the CCK-8 proliferation assay (Fig.2A), cell scratch assay (Fig.2B andC), and Transwell assay (Fig.2D andE) showed that C1QTNF1-AS1 knockdown promoted the proliferation, migration, and invasion of MG63 and U2OS cells. The Warburg effect plays a crucial role in tumor development and contributes to the growth of cancer cells.^28^ We explored whether C1QTNF1-AS1 is related to the Warburg effect in the development of OS by examining the glucose content and the amount of ATP generated in the supernatants of OS cell lines. We found that C1QTNF1-AS1 knockdown markedly increased the amount of ATP generated in MG63 and U2OS cells, but significantly reduced MG63 and U2OS cells the glucose content in the supernatant (Fig.2F) (indicating that OS cells consumed more glucose), thus promoting Warburg effector energy metabolism. Taken together, these data suggest that silencing C1QTNF1-AS1 promotes aerobic glycolysis in OS cells.

**3.3** **miR-34a-5p was identified as a direct target of C1QTNF1-AS1 and showed consistent expression trends in OS cells**

To explore the potential mechanism by which C1QTNF1-AS1 regulates aerobic glycolysis in OS, we used three target gene prediction algorithms, TarBase, miRDB, and TargetScan, to screen for C1QTNF1-AS1-targeted mRNA candidates. Intersection of the three databases revealed that only miR-34a-5p was associated with aerobic glycolysis(Fig.3A). The binding site of C1QTNF1-AS1 to miR-34a-5p was predicted according to the RNAhybrid and miRanda algorithms, and a mutated sequence, mut, was designed. The dual-luciferase assay results showed that the miR-34a-5p mimic could bind to the C1QTNF1-AS1 wild-type to increase luciferase activity compared with the expression of the NC mimic. However, when the binding site was mutated, the miR-34a-5p mimic had no significant effect on luciferase activity(Fig.3B). This indicates that miR-34a-5p binds to C1QTNF1-AS1 at this site(Fig.3C). To further explore the relationship between C1QTNF1-AS1 and miR-34a-5p in OS cells, we established a stable cell line (si-lnc) and a negative control (si-NC), and results of qRT-PCR experiments showed that miR-34a-5p expression in OS cells after silencing C1QTNF1-AS1 was also relatively reduced (Fig.3D).

**3.4** **miR-34a-5p inhibited the occurrence and development of OS cells by regulating the Warburg effect**

To further explore the role of miR-34a-5p in OS, stable cell lines (miR-mim) overexpressing miR and a negative control (mim-NC) were established and subjected to the CCK-8 proliferation assay. Results showed that overexpression of miR-34a-5p inhibited OS cell proliferative ability (Fig.4A) . Results of the Transwell assay showed that the overexpression of miR-34a-5p inhibited OS cell invasion (Fig.4B and C).Results of the scratch assay showed that the overexpression of miR-34a-5p inhibited OS cell migration (Fig.4D andE). We also found that overexpression of miR-34a-5p significantly inhibited the amount of ATP generated in MG63 and U2OS cell lines but significantly increased the glucose content in the cell supernatant (indicating that OS cells consumed less glucose) (Fig.4F), and thus, inhibited the Warburg effect. In conclusion, miR-34a-5p inhibited invasion, migration, and proliferative influences as well as aerobic glycolysis in OS cells.

**3.5** **Silencing of C1QTNF1-AS1 promoted OS progression through miR-34-a-5p–mediated glycolysis**

We further verified whether C1QTNF1-AS1 inhibits OS cell development and progression by targeting miR-34a-5p. Rescue experiments were performed using the U2OS and MG63 cell lines. By comparing results of the CCK-8（Fig5A）, Transwell（Fig5B andC）, and cell scratch assays, we found that silencing C1QTNF1-AS1 significantly promoted the proliferation, migration, and invasion of OS cells, and the overexpression of miR-34a-5p partially reversed this effect (Fig.5D andE). Similarly, the overexpression of miR-34a-5p partially reversed the effect of C1QTNF1-AS1 silencing on the Warburg effect in OS cells (Fig.5F). Together, these findings suggest that silencing C1QTNF1-AS1 promotes the proliferation, migration, and invasion of OS cells by inhibiting miR-34a-5p–mediated glycolysis.

**3.6 LDHA and PDK3 were identified as a direct targets of miR-34a-5p and showed opposite expression trends in OS cells**

The binding sites of LDHA, PDK3, and miR-34a-5p were predicted according to TargetScan, miRanda, and miRWalk algorithms, and the mutated sequence, mut, was designed. Results of the dual luciferase assay showed that the miR-34a-5p mimic binds to LDHA and PDK3 wild-type to reduce luciferase activity compared with the expression of NC mimic. However, when the binding site was mutated, the miR-34a-5p mimic had no significant effect on the luciferase activity. (Fig.6A andB) shows that miR-34a-5p binds to LDHA and PDK3 through this site(Fig.6C andD).Stable cell lines overexpressing miR-34a-5p (miR-mim) and a negative control (mim-NC) were established. PDK3 expression in OS cells significantly decreased, as did that of LDHA, after miR-34a-5p treatment , as determined by qRT-PCR and western blotting (Fig.6E-G).

**3.7** **Silencing of C1QTNF1-AS1 upregulates LDHA and PDK3 expression in OS cells through inhibition of miR-34a-5p**

To explore the interaction between C1QTNF1-AS1, miR-34a-5p, LDHA, and PDK3 in OS cells, we constructed C1QTNF1-AS1 knockdown stable cell lines (si-lnc) and miR-34a-5p overexpression stable cell lines (miR-mim). Results of RT-PCR and western blotting revealed that the expression of LDHA and PDK3 in OS cells increased significantly after C1QTNF1-AS1 silencing (Fig.7A-C).Through rescue experiments, we found that the overexpression of miR-34a-5p partially reversed the promoting effect of LDHA and PDK3 after silencing C1QTNF1-AS1 (Fig.7D-F). Mechanism diagram of LncRNA C1QTNF1-AS1 in regulating LDHA / PDK 3-mediated Warburg effect in osteosarcoma (Fig.7G).

**4. Discussion**

Osteosarcoma, as a common clinical primary bone tumor, mostly occurs in adolescents or children, and its incidence ranks first among primary bone malignant tumors.^29^ Mainly characterized by rapid progression and easy metastasis, it is a highly malignant bone tumor.^30^ Studies suggest that lncRNA plays a crucial role in the progression of various cancers.^31,32^ In the present study, we focused on the mechanism and function of lncRNA C1QTNF1-AS1 in OS. The results showed that silencing C1QTNF1-AS1 promoted the expression of LDHA and PDK3 via the adsorption of miR-34a-5p.

Increasing evidence suggests that dysregulation of C1QTNF1-AS1 is associated with many tumor progression processes, including in OS.^21,33^ Previous studies have shown that C1QTNF1-AS1 is significantly downregulated in some tumor cells and that C1QTNF1-AS1 suppresses a variety of cancers (e.g., colorectal, ovarian, pancreatic, gastric, and hepatocellular carcinoma) including proliferation, invasion, epithelial-interstitial transformation, and induction of apoptosis.^20,32,34,35^ C1QTNF1-AS1 has been found to inhibit the Warburg effect in colorectal cancer.^36^ In the present study, we validated the role of C1QTNF1-AS1 in OS cells. Functional experiments showed that silencing C1QTNF1-AS1 promoted proliferation, migration, invasion, and the Warburg effect in OS cells. Taken together, these results suggest that C1QTNF1-AS1 plays a crucial role in OS progression.

Several studies have demonstrated that miR-34a-5p can suppress malignancy in the cervix by inhibiting cell proliferation and invasion.^37^ The development of HNSCC was inhibited by miR-34a-5p by targeting Flotillin-2.^27^ In the present study, miR-34a-5p was predicted to be a C1QTNF1-AS1 target, and this interaction was validated by a dual-luciferase reporter assay. We experimentally verified a positive correlation between miR-34a-5p and C1QTNF1-AS1 expression trends in OS cells. Subsequently, using functional experiments, we verified the inhibition of cell proliferation, migration, invasion, and the Warburg effect in OS cells following miR-34a-5p overexpression. Furthermore, the effect of C1QTNF1-AS1 silencing was demonstrated by rescue experiments which resulted in the promotion of OS cell proliferation, invasion, migration, and Warburg effect by the adsorption of miR-34a-5p. These data showed that C1QTNF1-AS1 regulates OS progression by adsorbing miR-34a-5p.

Numerous studies have confirmed the existence of a Warburg effect in malignant tumor cells and demonstrated that it has an important influence on tumor development and progression.^38^ LDHA, a key enzyme in the last step of the Warburg effect, is highly expressed in many tumor cells and is closely correlated with tumor size and prognosis.^39,40^ PDK, having four subtypes (PDK1, PDK2, PDK3, PDK4), is closely related to the generation of the Warburg effect and is centrally positioned at the crossroads of glycolysis and oxidative phosphorylation.^41,42^ In the present study, we predicted LDHA and PDK3 as direct targets of miR-34a-5p, followed by verification by a dual-luciferase reporter gene assay. Moreover, the expression levels of C1QTNF1-AS1, miR-34a-5p, LDHA, and PDK3 in OS cells and rescue experiments showed that silencing C1QTNF1-AS1 upregulated LDHA and PDK3 expression levels in OS cells via adsorption of miR-34a-5p.

In conclusion, these results indicate that silencing lncRNA C1QTNF1-AS1 provides potential therapeutic targets for osteosarcoma treatment by inhibiting the expression of miR-34a-5p, thus promoting the expression of LDHA and PDK3 in OS cells and promoting the development of osteosarcoma by regulating the Warburg effect.

**Declarations**

**Ethics approval and consent to participate**：

This study was approved by the Ethics Committee of the Affiliated Hospital of Chengde Medical College. The ethics number is the CYFYLL2022106.

**Consent for publication**：

All authors have read and agreed to the published version of the manuscript.

**Availability of data and materials：**

The data supporting the results of this study can be obtained from the first author upon request. AND all datasets presented in this study are included in the article/Supplementary Materials

**Competing interests**：

The authors declare no competing interests.

**Funding**：

This study was supported by National Natural Science Foundation of China (NO. 82305055), Medical Science Research Project Program of Hebei Provincial Health Commission (No. 20210121), and Hebei Natural Science Foundation (H2022406038).

**Authors' contributions** :

Naiqiang Zhu designed the experimental scheme of this project. Yu Zhang completed all the experiments in this project and drafted the manuscript. Hailong Lun, Ning Yang, Kaikai Ding, Jialu Wang provided technical services and manuscript revision for this project, Bin Chen, Chengbing Chang, Yanqi Liu and Haipeng Gu performed the material preparation and data analysis. all authors commented on previous versions of the manuscript. All authors read and approved the final manuscript.

**Acknowledgements**：

Thank to all the teachers in the Central Laboratory of the Affiliated Hospital of Chengde Medical College for their contributions to this research。

**Abbreviations**

miR: microRNA

lncRNA: long noncoding RNA

OS: osteosarcoma

si: knockdown

mim: overexpression

LDHA: Lactate Dehydrogenase A

PDK3: Pyruvate Dehydrogenase Kinase 3

NC: negative control

**Figure legend**

**Fig. 1.** Expression of LncRNA‐C1QTNF1-AS1 is decreased in osteosarcoma (OS). Volcano plot illustrating the differentially expressed LncRNA in GEO datasets ([GSE42352](https://www.ncbi.nlm.nih.gov/geo/query/acc.cgi?acc=GSE65071)). (A) Heatmap showing the differentially expressed LncRNA in [GSE42352](https://www.ncbi.nlm.nih.gov/geo/query/acc.cgi?acc=GSE65071) obtained from the GEO database. (B) Expression of LncRNA C1QTNF1-AS1 in normal cells and OS cells of [GSE42352](https://www.ncbi.nlm.nih.gov/geo/query/acc.cgi?acc=GSE65071) dataset. (C) LncRNA C1QTNF1-AS1 levels of U2OS, Saos2, MG63, and HOS relative to hFOB1.19 cells were determined using quantitative real‐time PCR（D）. Results are presented as mean ± SD. **p* < 0.05, ****p* < 0.001.

**Fig. 2.** LncRNA‐C1QTNF1-AS1 suppresses osteosarcoma (OS) progression in vitro. CCK‐8 assay was used to assess the cell proliferation rate of OS cells after C1QTNF1-AS1 knockdown. (A) Cell scratch assays were used to evaluate the migration capacity of C1QTNF1-AS1 knockdown. (B,C)Transwell assays were used to evaluate the Invasion capacity of C1QTNF1-AS1 knockdown(D,E) The cell ATP levels assays were used to evaluate the ATP production level of C1QTNF1-AS1 knockdown and the Glucose content assays were used to evaluate the amount of glucose in the cell supernatant of C1QTNF1-AS1 knockdown.(F).Results are presented as mean ± SD. P<0.05,**P<0.01***P<0.001

**Fig.3.**miR-34a-5p is an authentic target of LncRNA‐C1QTNF1-AS1. The venn diagram showing the predicted glycolysis‐related target genes of LncRNA C1QTNF1-AS1 obtained from TarBase，miRDB，TargetScan databases. (A) Dual‐luciferase reporter assays in OS cells transfected with miR-34a-5p UTR WT or mutant (MUT) in combination with LncRNA C1QTNF1-AS1 plasmid or a negative control plasmid.(B) miR-34a-5p 3′‐UTR contains one predicted LncRNA C1QTNF1-AS1 binding site. (C) miR-34a-5p expression in osteosarcoma (OS) cells transfected with a negative control or C1QTNF1-AS1 knockdown plasmid. (D) Results are presented as mean ± SD. ***p* < 0.01, ****p* < 0.001. ns, *p* > 0.05.

**Fig.4.** miR-34a-5p suppresses osteosarcoma (OS) progression in vitro. CCK‐8 assay was used to assess the cell proliferation rate of OS cells after miR-34a-5p overexpression. (A) Transwell assays were used to evaluate the Invasion capacity of miR-34a-5p overexpression. (B,C) Cell scratch assays were used to evaluate the migration capacity of miR-34a-5p overexpression. (D,E) The cell ATP levels assays were used to evaluate the ATP production level of miR-34a-5p overexpression and the Glucose content assays were used to evaluate the amount of glucose in the cell supernatant of miR-34a-5p overexpression.(F). Results are presented as mean ± SD. P<0.05,**P<0.01***P<0.001

**Fig 5.** Overexpression of miR-34a-5p partly recovers the oncogenic function of C1QTNF1-AS1 knockdown in osteosarcoma (OS). According to the CCK‐8 assay（A），Transwell assays（B,C）and Cell scratch assays（D,E）indicate the overexpression of miR-34a-5p partially reversed the promoting effect of C1QTNF1-AS1 silencing on the proliferation, invasion and migration of OS cells. According to the cell ATP levels assays and the Glucose content assays (F) shown that miR-34a-5p overexpression partially reversed the effect of silencing C1QTNF1-AS1 on the Warburg effect in OS cells

**Fig.6.** LDHA and PDK3 is an authentic target of miR-34a-5p. Dual‐luciferase reporter assays in OS cells transfected with LDHA and PDK3 UTR WT or mutant (MUT) in combination with miR-34a-5p plasmid or a negative control plasmid. (A, B) LDHA and PDK3 3′-UTR each Contains a predicted miR-34a-5p binding site (C, D). LDHA and PDK3 expression in osteosarcoma (OS) cells transfected with a negative control or miR-34a-5p overexpression plasmid. (E-G) Results are presented as mean ± SD. ***p* < 0.01, ****p* < 0.001. ns, *p* > 0.05.

**Fig. 7.** Silencing of C1QTNF1-AS1 upregulates the expression of LDHA, PDK3 in OS cells through inhibition of miR-34a-5p. LDHA, PDK3 expression in OS cells after silencing of C1QTNF1-AS1. (A-C) miR-34a-5p overexpression could partially reverse the promoting effect of LDHA, PDK3 expression after silencing C1QTNF1-AS1(D-F)

Mechanism of LncRNA C1QTNF1-AS1 multitarget regulation of LDHA / PDK3-mediated Warburg effects in osteosarcoma Fig（G）

**References**:

1. Corre, I., Verrecchia, F., Crenn, V., Redini, F. & Trichet, V. The Osteosarcoma Microenvironment: A Complex but Targetable Ecosystem. *Cells* **9**, 976 (2020).

2. Shen, Y. *et al.* LncRNA KCNQ1OT1 sponges miR-34c-5p to promote osteosarcoma growth via ALDOA enhanced aerobic glycolysis. *Cell Death Dis.* **11**, 278 (2020).

3. Pan, X. *et al.* miR-1297 Suppresses Osteosarcoma Proliferation and Aerobic Glycolysis by Regulating PFKFB2. *OncoTargets and therapy* **13**, 11265–11275 (2020).

4. Lu, J. *et al.* IRX1 hypomethylation promotes osteosarcoma metastasis via induction of CXCL14/NF-κB signaling. *J. Clin. Invest.* **125**, 1839–1856 (2015).

5. Weng, Y. *et al.* MicroRNA‐324‐3p inhibits osteosarcoma progression by suppressing PGAM1‐mediated aerobic glycolysis. *Cancer Sci.* **114**, 2345–2359 (2023).

6. Chelakkot, C., Chelakkot, V. S., Shin, Y. & Song, K. Modulating Glycolysis to Improve Cancer Therapy. *International Journal of Molecular Sciences* **24**, 2606 (2023).

7. Zhong, X. *et al.* Warburg effect in colorectal cancer: the emerging roles in tumor microenvironment and therapeutic implications. *J. Hematol. Oncol.* **15**, 160 (2022).

8. Atas, E., Oberhuber, M. & Kenner, L. The Implications of PDK1–4 on Tumor Energy Metabolism, Aggressiveness and Therapy Resistance. *Frontiers in Oncology* **10**, (2020).

9. Pathria, G. *et al.* Targeting the Warburg effect via LDHA inhibition engages ATF4 signaling for cancer cell survival. *Embo J.* **37**, e99735 (2018).

10. Xu, K. *et al.* Glycolysis Fuels Phosphoinositide 3-Kinase Signaling to Bolster T Cell Immunity. *Science (New York, N.Y.)* **371**, 405–410 (2021).

11. Fang, Y. *et al.* RBBP7, regulated by SP1, enhances the Warburg effect to facilitate the proliferation of hepatocellular carcinoma cells via PI3K/AKT signaling. *J Transl Med* **22**, 170 (2024).

12. Yang, M. *et al.* A novel signature to guide osteosarcoma prognosis and immune microenvironment: Cuproptosis-related lncRNA. *Front. Immunol.* **13**, 919231 (2022).

13. Xing, C., Sun, S., Yue, Z.-Q. & Bai, F. Role of lncRNA LUCAT1 in cancer. *Biomedicine & Pharmacotherapy* **134**, 111158 (2021).

14. Chi, Y., Wang, D., Wang, J., Yu, W. & Yang, J. Long Non-Coding RNA in the Pathogenesis of Cancers. *Cells* **8**, 1015 (2019).

15. Liu, J., Feng, G., Li, Z., Li, R. & Xia, P. Long Non-Coding RNA FEZF1-AS1 Modulates CXCR4 to Promote Cell Proliferation, Warburg Effect and Suppress Cell Apoptosis in Osteosarcoma by Sponging miR-144. *OncoTargets and therapy* **13**, 2899–2910 (2020).

16. Pu, F. *et al.* LncCCAT1 interaction protein PKM2 upregulates SREBP2 phosphorylation to promote osteosarcoma tumorigenesis by enhancing the Warburg effect and lipogenesis. *Int. J. Oncol.* **60**, 44 (2022).

17. Hajibabaei, S. *et al.* Targeting long non-coding RNA MALAT1 reverses cancerous phenotypes of breast cancer cells through microRNA-561-3p/TOP2A axis. *Sci. Rep.* **13**, 8652 (2023).

18. Xiu, B. *et al.* LINC02273 drives breast cancer metastasis by epigenetically increasing AGR2 transcription. *Mol Cancer* **18**, 187 (2019).

19. Shen, Y. *et al.* Comprehensive Analysis of Tumor Immune Microenvironment and Prognosis of m6A-Related IncRNAs in Lung Adenocarcinoma. *Crit. Rev. Eukaryot. Gene Expr.* **32**, 77–91 (2022).

20. Schwerdtfeger, M. *et al.* Long non-coding RNAs in cancer stem cells. *Transl. Oncol.* **14**, 101134 (2021).

21. Han, W. *et al.* Potential of C1QTNF1-AS1 regulation in human hepatocellular carcinoma. *Mol. Cell. Biochem.* **460**, 37–51 (2019).

22. Hua, Q. *et al.* LINC01123, a c-Myc-activated long non-coding RNA, promotes proliferation and aerobic glycolysis of non-small cell lung cancer through miR-199a-5p/c-Myc axis. *J Hematol Oncol* **12**, 91 (2019).

23. Kousar, K. *et al.* miRNAs in Regulation of Tumor Microenvironment, Chemotherapy Resistance, Immunotherapy Modulation and miRNA Therapeutics in Cancer. *International Journal of Molecular Sciences* **23**, 13822 (2022).

24. Celik, B., Cicek, K., Leal, A. F. & Tomatsu, S. Regulation of Molecular Targets in Osteosarcoma Treatment. *International Journal of Molecular Sciences* **23**, 12583 (2022).

25. Jiang, M. *et al.* Exosome-mediated miR-144-3p promotes ferroptosis to inhibit osteosarcoma proliferation, migration, and invasion through regulating ZEB1. *Mol. Cancer* **22**, 113 (2023).

26. Pu, Y., Zhao, F., Wang, H. & Cai, S. MiR-34a-5p promotes multi-chemoresistance of osteosarcoma through down-regulation of the DLL1 gene. *Sci. Rep.* **7**, 44218 (2017).

27. Li, X. *et al.* miR-34a-5p functions as a tumor suppressor in head and neck squamous cell cancer progression by targeting Flotillin-2. *Int J Biol Sci* **17**, 4327–4339 (2021).

28. Halma, M. T. J., Tuszynski, J. A. & Marik, P. E. Cancer Metabolism as a Therapeutic Target and Review of Interventions. *Nutrients* **15**, 4245 (2023).

29. Bian, J. *et al.* Research progress in the mechanism and treatment of osteosarcoma. *Chin Med J (Engl)* **136**, 2412–2420 (2023).

30. Shoaib, Z., Fan, T. M. & Irudayaraj, J. M. K. Osteosarcoma mechanobiology and therapeutic targets. *Br J Pharmacol* **179**, 201–217 (2022).

31. Yu, X. *et al.* LncRNA‐HOTAIRM1 promotes aerobic glycolysis and proliferation in osteosarcoma via the miR‐664b‐3p/Rheb/mTOR pathway. *Cancer Sci.* **114**, 3537–3552 (2023).

32. Park, E.-G., Pyo, S.-J., Cui, Y., Yoon, S.-H. & Nam, J.-W. Tumor immune microenvironment lncRNAs. *Brief. Bioinform.* **23**, bbab504 (2021).

33. Cagle, P., Qi, Q., Niture, S. & Kumar, D. KCNQ1OT1: An Oncogenic Long Noncoding RNA. *Biomolecules* **11**, 1602 (2021).

34. Tan, Y. *et al.* LncRNA‐mediated posttranslational modifications and reprogramming of energy metabolism in cancer. *Cancer Commun.* **41**, 109–120 (2020).

35. Huang, Z., Zhou, J.-K., Peng, Y., He, W. & Huang, C. The role of long noncoding RNAs in hepatocellular carcinoma. *Mol Cancer* **19**, 77 (2020).

36. Park, M. K. *et al.* NEAT1 Is Essential for Metabolic Changes that Promote Breast Cancer Growth and Metastasis. *Cell Metab* **33**, 2380-2397.e9 (2021).

37. Jiang, T. & Cheng, H. miR-34a-5p blocks cervical cancer growth and migration by downregulating CDC25A.

38. Stine, Z. E., Schug, Z. T., Salvino, J. M. & Dang, C. V. Targeting cancer metabolism in the era of precision oncology. *Nat. Rev. Drug Discov.* **21**, 141–162 (2022).

39. Xia, P. *et al.* METTL5 stabilizes c‐Myc by facilitating USP5 translation to reprogram glucose metabolism and promote hepatocellular carcinoma progression. *Cancer Commun.* **43**, 338–364 (2023).

40. Jing, Z. *et al.* NCAPD3 enhances Warburg effect through c-myc and E2F1 and promotes the occurrence and progression of colorectal cancer. *J Exp Clin Cancer Res* **41**, 198 (2022).

41. Sun, W.-H., Chen, Y.-H., Lee, H.-H., Tang, Y.-W. & Sun, K.-H. PDK1- and PDK2-mediated metabolic reprogramming contributes to the TGFβ1-promoted stem-like properties in head and neck cancer. *Cancer Metab* **10**, 23 (2022).

42. Luengo, A. *et al.* Increased demand for NAD+ relative to ATP drives aerobic glycolysis. *Molecular cell* **81**, 691-707.e6 (2021).
