# Supplementary material for: Deciphering the oncogenic network: how C1QTNF1-AS1 modulates osteosarcoma through miR-34a-5p and glycolytic pathways
Source: Front Oncol. 2025 Jan 9;14:1485605. doi: 10.3389/fonc.2024.1485605 (PMC11754200; doi:10.3389/fonc.2024.1485605)
Supplement: Supplementary file 22 [file Table7.doc]

**Silencing of C1QTNF1-AS1 regulates the LDHA / pdk3-mediated warburg effect in osteosarcoma through adsorption of miR-34a-5p**

**Yu Zhang^ab^, Hailong Lun ^c^，Naiqiang Zhu^a*^, Ning Yang^b^, Kaikai Ding^ab^, Bin Chen^a^, Chengbing Chang^a^, Haipeng Gu^a^, Yanqi Liu^a^**

a Department of Minimally Invasive Spine Surgery, Affiliated Hospital of Chengde Medical University, Chengde, Hebei Province, China

b Hebei Key Laboratory of Panvascular Diseases, Chengde, Hebei, China

c Tangshan Nanhu Hospital, Tangshan, Hebei, China

**Corresponding Author:**

Naiqiang Zhu*, Division of Minimally Invasive Spine Surgery, Chengde Medical University Affiliated Hospital, 36 Nanyingzi St., Chengde, Hebei, 067000, PRC

Email address: [zhunq2010@163.com](mailto:zhunq2010@163.com)

**Abstract**

**Background**

Osteosarcoma (OS), a frequently encountered metastatic cancer in youth, typically carries a poor prognosis. Long non-coding RNAs (lncRNAs), including C1QTNF1-AS1, play a vital role in regulating cancer cell growth and motility. Known as an oncogene, C1QTNF1-AS1 is implicated in various tumor types, such as colorectal, pancreatic, and hepatocellular carcinomas, as well as OS. The current study delved into the functions and mechanisms of C1QTNF1-AS1 in the context of osteosarcoma.

**Methods**

In this investigation, we centered our attention on elucidating the functional roles and underlying mechanisms of C1QTNF1-AS1 in OS cells. Leveraging bioinformatics tools, we uncovered that microRNA miR-34a-5p specifically interacts with C1QTNF1-AS1, while LDHA and PDK3 are also targeted by miR-34a-5p. This interaction was further validated using dual-luciferase reporter assays. We analyzed the expression profiles of C1QTNF1-AS1, miR-34a-5p, LDHA, and PDK3 in osteosarcoma cells employing RT-PCR and western blot methodologies, thereby unraveling their intricate relationships. Additionally, we assessed the impact of these molecules on OS cell proliferation, invasion, and migration through CCK-8, Transwell, and wound healing assays. Furthermore, we examined their effects on aerobic glycolysis in OS cells by quantifying ATP levels and glucose uptake capacity.

**Results**

The findings revealed a marked decrease in C1QTNF1-AS1 expression levels in OS cells compared to normal osteoblasts. Notably, miR-34a-5p displayed a parallel downregulation trend in OS cells. Upon C1QTNF1-AS1 silencing, a pronounced upregulation of LDHA and PDK3 was observed in OS cells, which was partially attenuated by miR-34a-5p mimics. Functional evaluations demonstrated that suppression of C1QTNF1-AS1 accelerated OS cell growth, motility, invasiveness, and the Warburg effect. Conversely, miR-34a-5p overexpression mitigated these stimulatory effects, suggesting a regulatory role in modulating OS progression.

**Conclusions**

In conclusion, our research underscores the crucial role of C1QTNF1-AS1 in osteosarcoma (OS) pathogenesis. We discovered that the downregulation of C1QTNF1-AS1 indirectly upregulates the expression of LDHA and PDK3 through suppression of miR-34a-5p, which acts as a regulator of the Warburg effect. This cascade of events promotes OS progression by enhancing glycolytic metabolism and fueling cancer cell growth, migration, and invasion.

**Keywords**: OS, C1QTNF1-AS1, miR-34a-5p, LDHA, PDK3, Warburg Effect.

**1. Introduction**

Osteosarcoma, commonly abbreviated as OS, represents a highly aggressive form of connective tissue malignancy characterized by the capability of cancerous cells to produce bone and osteoid tissues. ^1^ It is the leading primary malignancy affecting bone tissues. Posing significant challenges for treatment due to its aggressive nature and propensity for dissemination.^2^ OS continues to yield poor patient outcomes despite rigorous therapeutic interventions.^3^ Consequently, there is an urgent need to unravel the intricate molecular mechanisms underpinning OS development, identify novel biomarkers to facilitate early diagnosis, and explore innovative therapeutic strategies to address this formidable disease.

To elucidate the Warburg effect, it implies that cancer cells, under hypoxic conditions, favor lactate fermentation over oxidative phosphorylation for energy generation.^4^ This metabolic shift is marked by intensified glycolysis, augmented glucose consumption, escalated lactate excretion, and decreased oxygen utilization within tumor cells. ^5,6^Extensive research has confirmed the manifestation of the Warburg effect in cancer cells, emphasizing its pivotal contribution to tumor progression and expansion. This phenomenon underscores the metabolic reprogramming of tumor cells, pivotal for their survival and aggressive growth.

PDK3, or pyruvate dehydrogenase kinase isoform 3, serves as a key regulator of intracellular energy metabolism, particularly within tumor cells. ^7^By inhibiting pyruvate dehydrogenase activity, PDK3 exerts a pronounced influence on the metabolic profile of cancer cells. ^8^This enzyme plays a central role in perpetuating the Warburg effect, a phenomenon intimately tied to energy metabolism and tumor cell vitality. ^9^Meanwhile, lactate dehydrogenase A (LDHA), a pivotal player in intracellular lactate dynamics, is intricately linked to both glycolysis and the Warburg effect.^10^ As a gene and enzyme implicated in glycolysis, LDHA facilitates the generation and accumulation of lactate, thereby bolstering cell survival and proliferation. ^11^This metabolic pathway is fundamental to cellular energy procurement and, as previous investigations have attested, is a hallmark of osteosarcoma (OS), underscoring its critical role in the disease.

Long non-coding RNAs (lncRNAs), comprising RNA molecules longer than 200 nucleotides without protein-coding capability, pervade the human genome.^12^ These transcripts exert substantial influence on the initiation, progression, and development of tumors, either bolstering or retarding these processes.^13,14^ For example, FEZF1-AS1, through its interaction with miR-4443, modulates the NUPR1-axis, thereby fostering the development of osteosarcoma (OS). ^15^In breast cancer, augmented levels of C1QTNF1-AS1 perturb growth, invasion, and dissemination of cancer cells by regulating various signaling cascades, such as Wnt/β-catenin, PI3K/Akt, and NF-κB. ^16–19^Likewise, in lung cancer, high expression of C1QTNF1-AS1 orchestrates critical cellular functions, including proliferation, invasion, and angiogenesis, by targeting genes like EGFR, HIF-1α, and VEGF. ^20–22^However, the precise function of C1QTNF1-AS1 in OS remains an enigma, necessitating further investigation.

MicroRNAs (miRNAs), consisting of approximately 22 nucleotides, are single-stranded, non-coding RNAs that have garnered substantial research attention. ^23^miR-323a-3p, for instance, amplifies LDHA expression, thereby augmenting lactate generation and fostering metastatic and invasive capabilities in osteosarcoma. ^24^Conversely, miR-199b-3p retards the expansion of osteosarcoma (OS) by targeting PDK1.^25^Notably, miR-34a-5p exhibits abnormal expression patterns in OS cells, and its levels are intimately tied to cellular responsiveness, tumor stage, lung metastasis potential, and patient prognosis.^26,27^ ^28^

The study unveiled intriguing findings regarding the roles of C1QTNF1-AS1 and miR-34a-5p in osteosarcoma (OS) cells. Specifically, it was observed that both C1QTNF1-AS1 and miR-34a-5p exhibited reduced expression levels in OS cells compared to normal osteoblasts. Furthermore, silencing C1QTNF1-AS1 led to a marked enhancement in OS cell proliferation and the Warburg effect, a phenomenon wherein cancer cells preferentially utilize glycolysis for energy generation even in the presence of oxygen. Bioinformatics analysis illuminated a potential interaction between C1QTNF1-AS1 and miR-34a-5p, suggesting that they partially base pair. Moreover, it was hypothesized that LDHA and PDK3, enzymes implicated in glycolysis and energy metabolism, could be target genes of miR-34a-5p. This interaction was subsequently validated using dual-luciferase reporter assays. The study delved deeper into the underlying mechanisms by exploring whether suppressing C1QTNF1-AS1 indirectly elevated LDHA and PDK3 expression levels through downregulation of miR-34a-5p. This, in turn, was postulated to influence the Warburg effect and accelerate tumor progression.

The research provides valuable insights into the complex interplay between lncRNAs, miRNAs, and their target genes in modulating cancer cell behavior. By shedding light on the regulatory mechanisms governing glycolysis and energy metabolism in OS cells, this study opens new avenues for the development of targeted therapies aimed at disrupting these pathways and ultimately retarding tumor growth and progression.

**2. Methods**

**2.1 Cell culture and reagents**

Human osteosarcoma cell lines (including MG63, Saos-2, U20S, and HOS) and normal osteoblasts ( hFOB1.19) were obtained from the ATCC Cell Bank located in Manassas, Virginia, USA. These cells were cultivated in Eagle's medium supplemented with 10% fetal bovine serum sourced from Thermo Fisher Scientific (Manassas, VA, USA), along with 0.1% penicillin and 0.1% streptomycin procured from Invitrogen (Carlsbad, CA, USA). The cultivation was conducted at 37°C in an incubator environment maintained at 5% CO2.

**2.2 Real-time quantitative PCR (qRT-PCR)**

In accordance with the manufacturer's protocol, RNA extraction from osteosarcoma cell lines including MG63, Saos-2, U20S, and HOS was accomplished using the TRIzol reagent sourced from Thermo Fisher Scientific. Subsequently, the RNA samples were reverse transcribed into cDNA using the PrimeScript RT Kit provided by Takara. Gene expression levels were then quantified via RT-qPCR on an ABI7500 Quantitative PCR instrument from ABI Corporation, employing the SYBR Prex Ex Taq II Kit also from Takara. GAPDH served as the stable reference gene for normalization during the analysis.

**2.3 Western blotting**

Total protein extraction was carried out using RIPA buffer (Sigma) supplemented with a protease inhibitor from Roche. The extracted proteins were then resolved on a 12% SDS-PAGE gel. After electrophoresis, the proteins were transferred to membranes which were subsequently blocked with 5% skim milk for an hour. Overnight incubation with the primary antibody at 4°C was followed by rinsing the membranes with TBST. Subsequently, the membranes were incubated with secondary antibodies and washed again with TBST. Luminescence was detected using an ECL detection kit from Share-bio. For quantification, densitometric analysis of the immunoblotted proteins was conducted utilizing ImageJ software.

**2.4 Cell Count Kit-8 (CCK-8) test**

Following transfection, U2OS and MG63 cells were plated in 96-well plates at a concentration of 3,000 cells per well. Each well received 10μL of CCK-8 solution (Dojindo Molecular Technologies) and was incubated at 37°C with 5% CO2 for 0, 24, and 48 hours. The optical density at 450 nm was then determined using a BioTek microplate reader.The findings reflect three separate trials.

**2.5 Cell transfection**

Cells with C1QTNF1-AS1 knockdown (si-lnc) and those with a negative control (si-NC) were produced. GenePharma (Shanghai, China) supplied the miR-34a-5p mimic (miR-mim) along with its control (miR-NC). Lipofectamine 3000 reagent (Invitrogen) was utilized for transfection, following the guidelines provided by the manufacturer.

**2.6 Measurement of** **glucose metabolism and cellular ATP levels**

The rate of glucose metabolism was assessed with a colorimetric glucose uptake test kit (Sigma-Aldrich) following the provided guidelines. The cellular ATP concentration was determined with an ATP assay kit from Promega (Madison, WI, USA) following the provided guidelines. The bioluminescence was measured using a fluorescence photometer from Perkin Elmer, located in Waltham, Massachusetts, USA.ATP levels were calculated using a standard curve.

**2.7 Transwell assay**

A matrix-coated 24-well span chamber (8μm aperture) was prepared for cell invasion assays. U2OS and MG63 cells were incubated in a serum-free medium in the upper chamber. The lower chamber received medium with 10% FBS. Following a 48-hour incubation period, the cells that had migrated to the lower chamber were treated with methanol and stained using a 0.1% crystal violet solution. The invading cells were examined using an Olympus inverted microscope.

**2.8 Cell scratch assay**

Cell scratch tests were utilized to evaluate the movement of OS cell lines. Once the U2OS and MG63 cells achieved 90% confluence in 24-well plates, a sterile plastic tip was used to scrape the monolayer, followed by two washes with phosphate-buffered saline (PBS) to eliminate cell debris. The cells were then incubated in complete growth medium. Ultimately, the cells that moved to the damaged region were gathered at 0 and 24 hours following the initial scratch and examined using an inverted microscope (Olympus) for each injury. The relative distances of the cell scratches were analyzed using ImageJ software.

**2.9 Dual-luciferase activity measurement**

A dual-luciferase reporter assay was conducted by inserting either wild-type or mutant lncRNA-C1QTNF1-AS1/LDHA/PDK3 into the pmirGLO vector (Universal Biotech, China).MG63 cells were planted in 48-well plates at a concentration of 5×104 cells per well. For 48 hours, the luciferase reporter plasmid along with miR-34a-5p mimic or mimic-NC were introduced using Liposome 3000 (Invitrogen).The activities of firefly and Renilla luciferase were evaluated using a dual-luciferase reporter assay kit from Promega, USA. The activity of Firefly luciferase was adjusted relative to that of Renilla luciferase. Every test was conducted three times.

**2.10 Bioinformatics analysis**

The dataset GSE42352 was sourced from the Gene Expression Omnibus (GEO) database, accessible at http://www.ncbi.nlm.nih.gov/geo.The downloaded data consisted of 84 disease samples and three healthy controls. The SangerBox platform (http://sangerbox.com) and the limma package (Linear Models for Microarray Data version 3.46.0 from Bioconductor) were utilized to examine the two sample groups.

**2.11. Database analysis**

Data on potential miR-34a-5p-target genes were obtained using the online software packages TarBase (https://dianalab.e-ce.uth.gr), miRDB (https://mirdb.org), and TargetScan (<https://www.targetscan.org>.). Based on analyses from RNAhybrid and miRanda, C1QTNF1-AS1 targets miR-34a-5p.Based on TargetScan, miRanda, and miRWalk prediction databases, LDHA and PDK3 were predicted to be targets of miR-34a-5p.

**2.12 Statistical analysis**

Data were analyzed and graphed using GraphPad Prism 9 (version 9.4.0).Data are presented as mean ± standard deviation, and group differences were analyzed using the t-test.A p-value of less than 0.05 was considered statistically significant.

**3.** **Results**

**3.1 C1QTNF1-AS1 expression was significantly downregulated in OS cells**

The SangerBox platform was utilized to analyze gene expression profiles from the GSE42352 dataset on GEO, aiming to pinpoint genes with differential expression in OS cells. Differentially expressed genes between OS cells and normal osteoblasts were identified using the limma package (log FC > 1 and p < 0.05) and volcano and heatmap plots were prepared in The R Project for Statistical Computing (R version 4.4.0)(Fig.1A and 1B).Among these differentially expressed genes, C1QTNF1-AS1 was significantly downregulated in OS cells compared to that in normal osteoblasts (Fig.1C).Initially, we assessed the expression levels of C1QTNF1-AS1 in both osteoblasts (hFOB 1.19) and various osteosarcoma cell lines (Saos-2, MG63, HOS, and U2OS) through qRT-PCR analysis. The findings indicated that C1QTNF1-AS1 expression was notably reduced in osteosarcoma cell lines relative to normal human osteogenic cell lines, with a marked decrease observed in MG63 and U2OS cell lines (Fig.1D).

**3.2 Silencing of C1QTNF1-AS1 significantly promoted OS cell development and the Warburg effect in vitro**

To investigate the precise role of C1QTNF1-AS1 in osteosarcoma cells, we created a cell line with C1QTNF1-AS1 knocked down (si-lnc) and a corresponding negative control (si-NC). The results of the CCK-8 proliferation assay (Fig.2A), wound healing assay (Fig.2B and 2C), along with the Transwell experiment (Fig.2D and 2E) demonstrated that reducing C1QTNF1-AS1 levels enhanced the growth, movement, and invasive capabilities of MG63 and U2OS cells. The Warburg effect is essential for tumor progression and aids in the proliferation of cancer cells. We investigated the potential link between C1QTNF1-AS1 and the Warburg effect in OS development by analyzing glucose levels and ATP production in the supernatants of OS cell cultures. Our research revealed that silencing C1QTNF1-AS1 led to a substantial rise in ATP production in MG63 and U2OS cells, while it notably decreased the glucose levels in the supernatant of these cells (Fig.2F), indicating that OS cells consumed more glucose and thus promoting Warburg effector energy metabolism. Taken together, these data suggest that silencing C1QTNF1-AS1 promotes aerobic glycolysis in OS cells.

**3.3 miR-34a-5p was identified as a direct target of C1QTNF1-AS1 and showed consistent expression trends in OS cells**

In order to investigate how C1QTNF1-AS1 influences aerobic glycolysis in OS, we employed three gene prediction tools—TarBase, miRDB, and TargetScan—to identify mRNA candidates targeted by C1QTNF1-AS1.Intersection of the three databases revealed that only miR-34a-5p was associated with aerobic glycolysis (Fig.3A).The binding site of C1QTNF1-AS1 to miR-34a-5p was predicted using the RNAhybrid and miRanda algorithms, and a mutated sequence, mut, was designed. The dual-luciferase assay results showed that the miR-34a-5p mimic could bind to C1QTNF1-AS1 wild-type to increase luciferase activity compared to the expression of the NC mimic. Nonetheless, altering the binding site resulted in the miR-34a-5p mimic showing no notable impact on luciferase activity (Fig.3B). This suggested that miR-34a-5p attaches to C1QTNF1-AS1 at this location (Fig.3C). In order to investigate the connection between C1QTNF1-AS1 and miR-34a-5p in osteosarcoma cells, we created a stable cell line (si-lnc) and a negative control (si-NC). The qRT-PCR analysis indicated that miR-34a-5p levels in osteosarcoma cells decreased following the silencing of C1QTNF1-AS1 (Fig.3D).

**3.4 miR-34a-5p suppressed the growth and progression of OS cells by modulating the Warburg effect.**

To investigate the function of miR-34a-5p in osteosarcoma, we created stable cell lines overexpressing miR (miR-mim) and a negative control (mim-NC), then performed CCK-8 proliferation tests. Elevated levels of miR-34a-5p suppressed the growth of OS cells (Fig.4A).The findings from the Transwell assay indicated that increased levels of miR-34a-5p suppressed the invasion of OS cells (Fig.4B and 4C).The scratch assay findings indicated that miR-34a-5p overexpression suppressed the migration of OS cells (Fig.4D and 4E).Our research revealed that miR-34a-5p overexpression markedly reduced ATP production in MG63 and U2OS cell lines, while notably elevating glucose levels in the cell supernatant, suggesting decreased glucose consumption by OS cells (Fig.4F), thereby inhibiting the Warburg effect. To sum up, miR-34a-5p suppresses the spread, movement, and growth effects, along with the aerobic glycolysis, of OS cells.

**3.5** **Silencing of C1QTNF1-AS1 promoted OS progression through miR-34-a-5p–mediated glycolysis**

We further verified whether C1QTNF1-AS1 inhibited OS cell development and progression by targeting miR-34a-5p. Rescue experiments were performed using the U2OS and MG63 cell lines. By comparing the results of the CCK-8 (Fig.5A), Transwell (Fig.5B and 5C)experiments and cell scratch tests.(Fig5D and 5E)we discovered that knocking down C1QTNF1-AS1 greatly enhanced the growth, movement, and infiltration of OS cells, while miR-34a-5p overexpression partially counteracted this outcome.In a similar manner, increasing the levels of miR-34a-5p somewhat counteracted the impact of C1QTNF1-AS1 suppression on the Warburg effect in osteosarcoma cells (Fig.5F).Overall, the results indicated that suppressing C1QTNF1-AS1 enhanced the growth, movement, and infiltration of OS cells by blocking miR-34a-5p–driven glycolysis.

**3.6** **LDHA and PDK3 were identified as direct targets of miR-34a-5p and showed opposite expression trends in OS cells**

The binding sites of LDHA, PDK3, and miR-34a-5p were predicted using the TargetScan, miRanda, and miRWalk algorithms, and the mutated sequence, mut, was designed. The dual-luciferase assay indicated that the miR-34a-5p mimic attached to the wild-type LDHA and PDK3, leading to a decrease in luciferase activity when compared to the NC mimic expression. Nonetheless, altering the binding site rendered the miR-34a-5p mimic ineffective in influencing luciferase activity.(Fig.6A and 6B) showed that miR-34a-5p binds to LDHA and PDK3 at this site (Fig.6C and 6D).Stable cell lines overexpressing miR-34a-5p (miR-mim) and negative control (mim-NC) were established.The levels of PDK3 and LDHA in osteosarcoma cells were notably reduced following miR-34a-5p administration, as evidenced by qRT-PCR and western blot analyses (Fig.6E-6G).

**3.7 Silencing of C1QTNF1-AS1 upregulates LDHA and PDK3 expression in OS cells through inhibition of miR-34a-5p**

To explore the interactions between C1QTNF1-AS1, miR-34a-5p, LDHA, and PDK3 in OS cells, we constructed C1QTNF1-AS1 knockdown stable cell lines (si-lnc) and miR-34a-5p overexpression stable cell lines (miR-mim).The suppression of C1QTNF1-AS1 in OS cells led to a notable rise in LDHA and PDK3 levels, as demonstrated by RT-PCR and western blot analyses (Fig.7A-7C).Our rescue experiments revealed that increasing miR-34a-5p levels somewhat counteracted the enhancement caused by LDHA and PDK3 following the knockdown of C1QTNF1-AS1 (Fig.7D-7F).The proposed mechanism of lncRNA C1QTNF1-AS1 in regulating the LDHA/PDK 3-mediated Warburg effect in osteosarcoma is shown in Fig.7G.

**4. Discussion**

Osteosarcoma (OS), a common form of primary bone malignancy encountered in clinical practice, predominantly strikes adolescents and children and boasts the highest incidence rate among its kind. Characterized by rapid progression and a high tendency for metastasis. ^29^OS is considered a highly aggressive cancer. In the realm of cancer development, long non-coding RNAs (lncRNAs) occupy a pivotal position.^30^This particular study delves into the functional roles and underlying mechanisms of the lncRNA, C1QTNF1-AS1, in the context of osteosarcoma. The findings of this investigation reveal that the inhibition or silencing of C1QTNF1-AS1 leads to an upregulation of LDHA and PDK3 expressions, a phenomenon mediated through the sequestration of miR-34a-5p. This observation underscores the intricate interplay between various regulatory elements within the cancer cell landscape.

The provided text discusses the role of C1QTNF1-AS1 in various types of cancer, including osteosarcoma (OS). It highlights that C1QTNF1-AS1 expression is reduced in certain cancer cells and that it inhibits multiple types of cancer by affecting proliferation, invasion, epithelial-mesenchymal transition, and apoptosis induction.^31,32^ Additionally, C1QTNF1-AS1 counteracts the Warburg effect in hepatocellular carcinoma.^33^The study confirms the function of C1QTNF1-AS1 in OS cells, showing that silencing C1QTNF1-AS1 promotes proliferation, migration, invasion, and the Warburg effect in OS cells. Overall, the findings indicate that C1QTNF1-AS1 is essential in the advancement of OS.

miR-34a-5p inhibits Thyroid cancer progression by restricting cell growth and spread^33^ and also hampers Head and neck squamous cell carcinoma development by targeting Flotillin-2. ^34^In this research, miR-34a-5p was identified as a target of C1QTNF1-AS1, confirmed through dual-luciferase reporter tests. Our analysis revealed a significant association between the levels of miR-34a-5p and C1QTNF1-AS1 in osteosarcoma (OS) cells. Functional assays confirmed that miR-34a-5p overexpression suppresses cell growth, movement, invasion, and the Warburg effect in OS cells. Rescue experiments showed that silencing C1QTNF1-AS1 led to increased OS cell growth, invasion, migration, and the Warburg effect by sequestering miR-34a-5p. These data indicate that C1QTNF1-AS1 regulates OS progression by adsorbing miR-34a-5p.

The Warburg effect plays a pivotal role in fostering the growth and progression of malignant tumors.^35^ Notably, lactate dehydrogenase A (LDHA), a pivotal enzyme in the final phase of this metabolic shift, is prevalent in multiple cancer cells and is intimately linked to tumor dimensions and clinical outcomes. ^36–38^Pyruvate dehydrogenase kinase (PDK), with its four isoforms, contributes significantly to the emergence of the Warburg effect and balances glycolysis with oxidative phosphorylation. ^39,40^Our study pinpointed LDHA and PDK3 as direct targets of microRNA-34a-5p (miR-34a-5p), validated through dual-luciferase reporter assays. Additionally, analyzing the expression patterns of C1QTNF1-AS1, miR-34a-5p, LDHA, and PDK3 in osteosarcoma (OS) cells, along with rescue experiments, revealed that the silencing of C1QTNF1-AS1 upregulates LDHA and PDK3 levels in OS cells by sequestering miR-34a-5p.

In summary, our research indicates that targeting the long non-coding RNA C1QTNF1-AS1 may pave the way for novel therapeutic strategies in osteosarcoma (OS) management. By inhibiting miR-34a-5p, this approach elevates LDHA and PDK3 levels in OS cells, modulating the Warburg effect and ultimately facilitating OS progression.

**Declarations**

Approval of ethics and agreement to join

The Ethics Committee at the Affiliated Hospital of Chengde Medical College granted approval for this research. The approval code from the ethics committee is CYFYLL2022106.

***Consent for publication***

Every author has reviewed and consented to the final version of the manuscript.

**Access to information and resources**

The first author can provide the data backing this study's findings upon request. The article and Supplementary Materials contain all the datasets discussed in this research.

***Competing interests***

The writers state that they have no conflicts of interest.

***Funding***

This study was supported by National Natural Science Foundation of China (NO. 82305055), Medical Science Research Project Program of Hebei Provincial Health Commission (No. 20210121), and Hebei Natural Science Foundation (H2022406038)

***Authors' contributions***

Naiqiang Zhu developed the experimental design for this project. Yu Zhang was responsible for conducting all experiments and drafting the manuscript. Technical support and manuscript editing were provided by Hailong Lun, Ning Yang, Kaikai Ding, and Jialu Wang. Bin Chen, Chengbing Chang, Yanqi Liu, and Haipeng Gu managed material preparation and data analysis. All authors reviewed earlier versions of the manuscript and approved the final draft.

***Acknowledgments***

We thank all the teachers at the Central Laboratory of the Affiliated Hospital of Chengde Medical College for their contributions to this study.

**Abbreviations**

miR: microRNA

lncRNA: long noncoding RNA

OS: osteosarcoma

si: knockdown

mim: overexpression

LDHA: lactate dehydrogenase A

PDK3, also known as pyruvate dehydrogenase kinase isoform 3

NC: negative control

**Figure legends**

Fig.1.The levels of LncRNA‐C1QTNF1-AS1 are reduced in osteosarcoma (OS).A volcano plot showcasing the LncRNA with differential expression in GEO datasets.(A) A heatmap illustrating the variably expressed LncRNA in GSE42352, sourced from the GEO database.(B) Expression of LncRNA C1QTNF1-AS1 in normal cells and OS cells of  dataset.(C) Quantitative real-time PCR (D) was employed to measure the levels of LncRNA C1QTNF1-AS1 in U2OS, Saos2, MG63, and HOS cells compared to hFOB1.19 cells. The outcomes are shown as the average ± standard deviation.* indicates p-value less than 0.05, while *** signifies p-value below 0.001.

Fig.2.LncRNA‐C1QTNF1-AS1 inhibits the advancement of osteosarcoma (OS) in laboratory settings. The CCK-8 test was employed to evaluate the growth rate of OS cells following the suppression of C1QTNF1-AS1.(A) The cell scratch test was employed to assess the migration ability following C1QTNF1-AS1 knockdown.(B,C) The invasive potential following C1QTNF1-AS1 suppression was assessed using Transwell assays (D,E).To assess ATP production in C1QTNF1-AS1 knockdown cells, ATP level assays were conducted, while glucose content in the cell supernatant was measured using glucose assays.(F) Results are shown as average ± standard deviation. P<0.05, **P<0.01, ***P<0.001.

Fig.3. miR-34a-5p is an authentic target of LncRNA‐C1QTNF1-AS1.A Venn diagram illustrating the anticipated glycolysis-associated target genes of lncRNA C1QTNF1-AS1,derived from the TarBase miRDB, and TargetScan databases.(A)Dual-luciferase reporter experiments were conducted on OS cells transfected with either miR-34a-5p UTR wild-type (WT) or mutant (MUT) forms, alongside lncRNA C1QTNF1-AS1 plasmid or a control plasmid.(B) miR-34a-5p 3′‐UTR contains one predicted LncRNA C1QTNF1-AS1 binding site.(C) Expression levels of miR-34a-5p in osteosarcoma (OS) cells after transfection with either a negative control or a C1QTNF1-AS1 knockdown plasmid.(D) Results are shown as the average ± standard deviation.**p < 0.01, ***p < 0.001. ns, p > 0.05.

Fig.4. miR-34a-5p inhibits the advancement of osteosarcoma (OS) in laboratory settings.CCK‐8 assay was used to assess the cell proliferation rate of OS cells after miR-34a-5p overexpression.(A) Transwell experiments were conducted to assess the invasive potential of miR-34a-5p overexpression.(B,C）To assess the migratory ability influenced by miR-34a-5p overexpression, scratch assays were conducted on cells.（D，E）To assess ATP production in cells overexpressing miR-34a-5p, ATP level assays were conducted, while glucose content in the cell supernatant was measured using glucose assays (F).The outcomes are shown as the average ± standard deviation.P<0.05, **P<0.01, ***P<0.001.

Fig.5.Elevated levels of miR-34a-5p partially restore the cancer-promoting role of C1QTNF1-AS1 suppression in osteosarcoma.CCK-8 tests (A), Transwell evaluations (B,C), and wound healing assays (D,E) indicate that miR-34a-5p overexpression somewhat counteracted the enhancement in OS cell proliferation, invasion, and migration caused by C1QTNF1-AS1 knockdown. Assays measuring ATP levels and glucose content (F) indicate that overexpressing miR-34a-5p somewhat counteracted the impact of C1QTNF1-AS1 silencing on the Warburg effect in osteosarcoma cells.

Fig.6. LDHA and PDK3 are authentic targets of miR-34a-5p. Dual-luciferase reporter experiments were conducted on OS cells transfected with either wild-type or mutant LDHA and PDK3 UTR, alongside miR-34a-5p plasmid or a control plasmid.（A,B）The 3′-UTRs of LDHA and PDK3 both have a predicted binding site for miR-34a-5p.（C,D）Expression levels of LDHA and PDK3 in osteosarcoma (OS) cells transfected with either a negative control plasmid or one overexpressing miR-34a-5p.（E-G）The findings are shown as the average ± standard deviation.**p < 0.01, ***p < 0.001. ns, p > 0.05.

Fig.7.Silencing of C1QTNF1-AS1 upregulates the expression of LDHA, PDK3 in OS cells through inhibition of miR-34a-5p. LDHA, PDK3 expression in OS cells after silencing of C1QTNF1-AS1.（A-C）Overexpressing miR-34a-5p somewhat counteracted the enhancement of LDHA and PDK3 levels following the knockdown of C1QTNF1-AS1.(D-F)

Fig.8 Mechanism of lncRNA C1QTNF1-AS1 multitarget regulation of LDHA/PDK3-mediated Warburg effects in osteosarcoma .

**References**

1. Yu, X. *et al.* LncRNA‐HOTAIRM1 promotes aerobic glycolysis and proliferation in osteosarcoma via the miR‐664b‐3p/Rheb/mTOR pathway. *Cancer Sci* **114**, 3537–3552 (2023).

2. Zheng, C. *et al.* The knockdown of lncRNA DLGAP1‐AS2 suppresses osteosarcoma progression by inhibiting aerobic glycolysis via the miR‐451a/HK2 axis. *Cancer Sci* **114**, 4747–4762 (2023).

3. ZHU, Y. *et al.* Hydroxysafflor yellow A induced ferroptosis of Osteosarcoma cancer cells by HIF-1α/HK2 and SLC7A11 pathway. *Oncol. Res.* **32**, 899–910.

4. Halma, M. T. J., Tuszynski, J. A. & Marik, P. E. Cancer Metabolism as a Therapeutic Target and Review of Interventions. *Nutrients* **15**, 4245 (2023).

5. Mathew, M., Nguyen, N. T., Bhutia, Y. D., Sivaprakasam, S. & Ganapathy, V. Metabolic Signature of Warburg Effect in Cancer: An Effective and Obligatory Interplay between Nutrient Transporters and Catabolic/Anabolic Pathways to Promote Tumor Growth. *Cancers (Basel)* **16**, 504 (2024).

6. Niepmann, M. Importance of Michaelis Constants for Cancer Cell Redox Balance and Lactate Secretion—Revisiting the Warburg Effect. *Cancers (Basel)* **16**, 2290 (2024).

7. Rai, S., Roy, G. & Hajam, Y. A. Melatonin: a modulator in metabolic rewiring in T-cell malignancies. *Front Oncol* **13**, 1248339 (2024).

8. Pucci, G. *et al.* Glut-3 Gene Knockdown as a Potential Strategy to Overcome Glioblastoma Radioresistance. *Int J Mol Sci* **25**, 2079 (2024).

9. Anwar, S., Shamsi, A., Mohammad, T., Islam, A. & Hassan, Md. I. Targeting pyruvate dehydrogenase kinase signaling in the development of effective cancer therapy. *Biochimica et Biophysica Acta (BBA) - Reviews on Cancer* **1876**, 188568 (2021).

10. Yang, W. *et al.* Cytoplasmic localization of SETDB1-induced Warburg effect via c-MYC-LDHA axis enhances migration and invasion in breast carcinoma. *Int J Mol Med* **53**, 40 (2024).

11. Lin, J. *et al.* Circular RNA circTATDN3 promotes the Warburg effect and proliferation in colorectal cancer. *Cancer Letters* **589**, 216825 (2024).

12. Liu, C. *et al.* LncRNA‐CCAT5‐mediated crosstalk between Wnt/β‐Catenin and STAT3 signaling suggests novel therapeutic approaches for metastatic gastric cancer with high Wnt activity. *Cancer Commun (Lond)* **44**, 76–100 (2023).

13. Li, L. *et al.* Role of LncRNA H19 in tumor progression and treatment. *Mol. Cell. Probes* **75**, 101961 (2024).

14. Wan, Q. *et al.* Tumor-infiltrating macrophage associated lncRNA signature in cutaneous melanoma: implications for diagnosis, prognosis, and immunotherapy. *Aging (Albany NY)* **16**, 4518–4540 (2024).

15. Zhou, C. *et al.* Long Noncoding RNA FEZF1-AS1 Promotes Osteosarcoma Progression by Regulating the miR-4443/NUPR1 Axis. *Oncol Res* **26**, 1335–1343 (2018).

16. Roles of lncRNA in the diagnosis and prognosis of triple-negative breast cancer. *J Zhejiang Univ Sci B* **24**, 1123–1140 (2023).

17. Cai, Z. *et al.* LncRNA EILA promotes CDK4/6 inhibitor resistance in breast cancer by stabilizing cyclin E1 protein. *Sci Adv* **9**, eadi3821.

18. Zhang, K.-J., Tan, X.-L. & Guo, L. LncRNA TYMSOS facilitates breast cancer metastasis and immune escape through downregulating ULBP3. *iScience* **26**, 107556 (2023).

19. Dong, Y., Zhang, T., Li, X., Yu, F. & Guo, Y. Comprehensive analysis of coexpressed long noncoding RNAs and genes in breast cancer. *Journal of Obstetrics and Gynaecology Research* **45**, 428–437 (2019).

20. Braga, E. A. *et al.* Various LncRNA Mechanisms in Gene Regulation Involving miRNAs or RNA-Binding Proteins in Non-Small-Cell Lung Cancer: Main Signaling Pathways and Networks. *International Journal of Molecular Sciences* **24**, (2023).

21. Yang, Q. *et al.* LINC02159 promotes non-small cell lung cancer progression via ALYREF/YAP1 signaling. *Mol. Cancer* **22**, 122 (2023).

22. Chen, Y.-L. *et al.* LncRNA SLCO4A1-AS1 suppresses lung cancer progression by sequestering the TOX4-NTSR1 signaling axis. *J. Biomed. Sci.* **30**, 80 (2023).

23. Yang, J. *et al.* MicroRNA-488: A miRNA with diverse roles and clinical applications in cancer and other human diseases. *Biomedicine & Pharmacotherapy* **165**, 115115 (2023).

24. Chen, H., Gao, S. & Cheng, C. MiR-323a-3p suppressed the glycolysis of osteosarcoma via targeting LDHA. *Human Cell* **31**, 300–309 (2018).

25. Zhu, D., Qi, H. & Zhu, H. hsa-miR-199b-3p suppresses osteosarcoma progression by targeting CCDC88A, inhibiting epithelial-to-mesenchymal transition, and Wnt/beta-catenin signaling pathway. *Sci Rep* **13**, 12544 (2023).

26. Zhang, Y., Mi, Y. & He, C. 2‐methoxyestradiol restrains non‐small cell lung cancer tumorigenesis through regulating circ_0010235/miR‐34a‐5p/NFAT5 axis. *Thorac Cancer* **14**, 2105–2115 (2023).

27. Dai, L. *et al.* circAGTPBP1 promotes the progression of papillary thyroid cancer through the notch pathway via the miR-34a-5p/notch1 axis. *iScience* **26**, 107564 (2023).

28. Chen, S. *et al.* MiR-34a-5p suppresses cutaneous squamous cell carcinoma progression by targeting SIRT6. *Arch Dermatol Res* **316**, 299 (2024).

29. Yang, Z. *et al.* LncRNA WAC-AS1 promotes osteosarcoma Metastasis and stemness by sponging miR-5047 to upregulate SOX2. *Biol. Direct* **18**, 74 (2023).

30. Wang, P. *et al.* LncRNA SATB2-AS1 promotes tumor growth and metastasis and affects the tumor immune microenvironment in osteosarcoma by regulating SATB2. *Journal of Bone Oncology* **41**, 100491 (2023).

31. Yu, H. *et al.* Dynamic network biomarker C1QTNF1 regulates tumor formation at the tipping point of hepatocellular carcinoma. *Biomolecules and Biomedicine* **24**, 939–951 (2024).

32. Qiu, J. *et al.* High C1QTNF1 expression mediated by potential ncRNAs is associated with poor prognosis and tumor immunity in kidney renal clear cell carcinoma. *Front Mol Biosci* **10**, 1201155 (2023).

33. Lin, J. C1QTNF1-AS1 regulates the occurrence and development of hepatocellular carcinoma by regulating miR-221-3p/SOCS3. *Hepatol Int* **15**, 526–526 (2021).

34. Dai, L. *et al.* circAGTPBP1 promotes the progression of papillary thyroid cancer through the notch pathway via the miR-34a-5p/notch1 axis. *iScience* **26**, 107564 (2023).

35. Lu, S. *et al.* Long non-coding RNA SNHG17 may function as a competitive endogenous RNA in diffuse large B-cell lymphoma progression by sponging miR-34a-5p. *PLOS ONE* **18**, e0294729 (2023).

36. Upadhyay, S., Khan, S. & Hassan, Md. I. Exploring the diverse role of pyruvate kinase M2 in cancer: Navigating beyond glycolysis and the Warburg effect. *Biochimica et Biophysica Acta (BBA) - Reviews on Cancer* **1879**, 189089 (2024).

37. Wu, C. *et al.* FOXQ1 promotes pancreatic cancer cell proliferation, tumor stemness, invasion and metastasis through regulation of LDHA-mediated aerobic glycolysis. *Cell Death Dis.* **14**, 699 (2023).

38. Chen, M. *et al.* Glycine Decarboxylase (GLDC) Plays a Crucial Role in Regulating Energy Metabolism, Invasion, Metastasis and Immune Escape for Prostate Cancer. *Int. J. Biol. Sci.* **19**, 4726–4743 (2023).

39. Yang, H. *et al.* Glucose transporter 3 (GLUT3) promotes lactylation modifications by regulating lactate dehydrogenase A (LDHA) in gastric cancer. *Cancer Cell Int.* **23**, (2023).

40. Gan, L. *et al.* Targeting the pyruvate dehydrogenase complex/pyruvate dehydrogenase kinase (PDC/PDK) axis to discover potent PDK inhibitors through structure-based virtual screening and pharmacological evaluation. *European Journal of Medicinal Chemistry* **264**, 116008 (2024).

41. Li, X.-N. *et al.* Formaldehyde induces ferroptosis in hippocampal neuronal cells by upregulation of the Warburg effect. *Toxicology* **448**, 152650 (2021).
